# *Acacia hydaspica* R. Parker ethyl-acetate extract abrogates cisplatin-induced nephrotoxicity by targeting ROS and inflammatory cytokines

**DOI:** 10.1038/s41598-021-96509-y

**Published:** 2021-08-26

**Authors:** Tayyaba Afsar, Suhail Razak, Dara Aldisi, Maria Shabbir, Ali Almajwal, Abdulaziz Abdullah Al Kheraif, Mohammed Arshad

**Affiliations:** 1 Department of Community Health Sciences, College of Applied Medical Sciences, King Saud University, Riyadh, Kingdom of Saudi Arabia; 2 Atta-Ur-Rahman School of Applied Biosciences, National University of Sciences and Technology, Islamabad, Pakistan; 3 Dental Biomaterials Research Chair, Dental Health Department, College of Applied Medical Sciences, King Saud University, Riyadh, Kingdom of Saudi Arabia

**Keywords:** Biochemistry, Cancer, Drug discovery, Nephrology

## Abstract

Cisplatin (CisPT) is a chemotherapeutic drug that outcomes in adverse effects. In this study, we examined the effect of *A. hydaspica* ethyl acetate extract (AHE) in an animal model of cisplatin-induced acute kidney injury (AKI). 36 male Sprague Dawley rats were used in the AKI rat model, and CisPT (7.5 mg/kg BW, i.p) single dose was given. In the pretreatment module, AHE (400 mg/kgBW/day, p.o) was given for 7 days before and after CisPT injection. While in the post-treatment group AHE was administered for 7 days after a single CisPT shot. The standard group received silymarin (100 mg/kg BW, p.o) for 7 days before and after CisPT injection. In HCT 116 tumor xenografts (*n* = 32) two groups of mice were pretreated with 400 mg/kg AHE orally for 7 days and two groups were treated with distilled water. On day 7 of pretreatment one distilled water and one AHE pretreated group were injected i.p with 15 mg/kg bw dose followed by another dose of CisPT 2 wk later. AHE groups were additionally treated with 400 mg/kg AHE for 3 days/week for 2 weeks. CisPT significantly deteriorated renal function parameters, i.e., PH, specific gravity, total protein, albumin, urea, creatinine, uric acid, globulin and blood urea nitrogen. CisPT treatment increased oxidative stress markers, while lower renal antioxidant enzymes. AHE pretreatment ameliorates significantly (*p* < 0.0001) CisPT-induced alterations in serum and urine markers for kidney function. Furthermore, AHE pretreatment more efficiently (*p* < 0.001) decreases oxidative stress markers, attenuate NF-κB, and IL-6 protein and mRNA expression by augmenting antioxidant enzyme levels compared to post-treatment. The histological observations verified the protective effect of AHE. In tumor xenograft mice, AHE treatment significantly reduced CisPT induced oxidative stress while it did not interfere with the anticancer efficacy of cisplatin as shown by significance (*p* < 0.001) decrease in tumor size after treatment. *A. hydaspica* AHE might provide a prospective adjuvant that precludes CisPT-induced nephrotoxicity without compromising its antitumor potential.

## Introduction

Cisplatin (cis diamminedichloroplatinum-II, CisPT) is a widely recommended medicine for the management of numerous cancers^1^. The major strife phase associated with CisPT chemotherapy is its resistance^2^ and adverse effects such as gastrointestinal toxicity, neurotoxicity, hepatotoxicity, and kidney toxicity. CisPT medical use is restricted as a result of its nephrotoxicity with about 25–35% of patients facing a substantial decline of kidney function after a single dose of CisPT treatment^3, 4^. Most of the DNA detrimental agents generally do not show as much toxicity in non-multiplying cells, yet CisPT selectively damages the non-dividing renal proximal tubule cells to a greater extent than in other organs^5^. Acute renal failure point to the abrupt and typically rescindable impairment of renal function. Besides the direct tubular toxicity, CisPT persuades dualistic cell death (apoptosis/necrosis). Necrosis has been generally allied with higher doses of CisPT, however apoptosis is concomitant with therapeutic doses. Also, inflammation has been implicated in the pathogenesis of CisPT-induced kidney toxicity. Several studies have testified that CisPT-induced oxidative stress is intricate in the instigation of renal tubule injury^3, 9^. CisPT induces reactive oxygen species (ROS) production such as superoxide anion and hydroxyl radicals conceivably due to the reduction of enzymatic and non-enzymatic antioxidant systems. Anomalous generation of ROS may result in impairment of macromolecules through various mechanisms, including peroxidation of membrane lipids, protein denaturation, DNA damage, inflammation, and apoptosis of normal cells, commencing oxidative renal dysfunction as a result of CisPT treatment. The contribution of oxidative trauma was additionally reinforced by the fact that free radical scavengers and antioxidants preclude CisPT-induced renal toxicity^6, 7^.

The current measures of nephron-protection used in patients receiving cisplatin are not adequate, and studies have dedicated to the research of new promising protective approaches. At present, several therapeutic strategies have been proposed as possible approaches for preventing CisPT-induced nephrotoxicity. However, clinical outcomes are still disappointing i.e., hydration therapy did not resolve renal dysfunction in a large number of treated patients^8^. Withdrawal of CisPT therapy remains the only decision for patients with advanced renal injury. Hence, finding effective approaches for precluding cisplatin-induced renal damage is a serious issue in cancer therapeutic exploration. Herbs and plant-based preparations have been used since epochs by herbal practitioners to treat human illnesses and decrease the effects of toxic constituents^9^. Hence, the leading goal of scientific exploration is to develop combinatorial therapies using compounds that have greater potency, minimum toxicity, and which did not compromise the anticancer efficacy of CisPT^2, 10^.

*Acacia hydaspica* R. Parker synonym; *Acacia eburnean* belongs to family Leguminosae. The vernacular name of the plant is Pahari Kikar, Kikar; Marmat. The bark and seeds are rich in tannins^11, 12^. We revealed that the principle bioactive components of *A. hydaspica* are 7-*O*-galloyl catechin, catechin, catechin-gallate and methyl gallate^13–15^, 1,2-Benzenedicarboxylic acid mono (2-Ethylhexyl) ester, α-Amyrin, Vitamin E, 2,6-dimethyl-*N*-(2-methyl-à-phenyl benzyl) aniline, and Squalene^16^ as a major antioxidant and anticancer composites from *A. hydaspica* ethyl acetate extract (AHE). In our previous investigations, we found that *A. hydaspica* possesses antioxidant, anticancer^13^ and anti-inflammatory^17^ potentials. Different species of genus *Acacia* were studied for their antioxidant and nephroprotective proficiencies in animal models^18^. The aqueous extract of *A. Senegal* showed significant nephroprotective potency against gentamicin (100 mg/kg) induced renal damage by lowering serum creatinine and urea levels in rats^19^. Administration of *A.nilotica* significantly inhibits cadmium chloride-induced decrease in serum albumin, globulin levels, albumin/globulin ratio, superoxide dismutase (SOD), and glutathione peroxidase (GPx) levels via ameliorating malondialdehyde(MDA) and nitric oxide (NO) content to protect renal damages^20^. Clinical trials are generally lacking. One trial used gum arabic (*A. senegal*) 30 g daily for 6 weeks as a dietary supplement to reduce weight. *Acacia* is essentially nontoxic when ingested and is generally recognized as safe (GRAS). In our previous lab investigations, we have identified that *A. hydaspica* inhibits CisPT induced liver^21^, heart^22^, pulmonary^23^ and testicular^24^ toxicity in a rodent model by preventing oxidative stress, ameliorating lipid profile, liver function tests (LFTs), cardiac function biomarkers and reproductive hormone alterations. Histological evidence also supports the role of *A. hydaspica* in managing CisPT side effects. Polyphenolic compounds are excellent drug candidates for preventing CisPT induced injuries. Previous studies reported that green tea polyphenols preclude CisPT-provoke nephrotoxicity in rat and polyphenols pretreatment was more significant than post-treatment in lessening CisPT-persuaded renal injury. Epigallocatechin gallate administration prevents CisPT induced renal damages, reduces oxidative kidney injuries and inflammative responses^25^. Epicatechin prevented the renal damage induced by lipopolysaccharides (LPS) by decreasing plasma creatinine and urea levels. Furthermore, epicatechin reduced expression of inflammatory molecules (TNF-α, iNOS, and IL-6), ameliorated NF-κB pathway, and attenuated superoxide anion creation and lipid peroxidation^26^. Silymarin, a flavonoid compound, is extracted from *Silybum marianum* seeds. Silymarin is well known herbal medicine to counter various ailments. Silymarin substantially interfere with numerous molecular mechanisms regulating cancer cell growth, progression, and angiogenesis. Silymarin combats cancer specified that it may offer hope not only for the inhibition of cancer growth, but also for the treatment of cancer, both alone and when combined with existing cancer drugs. This is because silymarin has shown direct tumor killing properties of its own, and is also synergistically effective with two popular chemotherapy agents, doxorubicin and cisplatin^17, 25, 26^. In current study we have used silymarin as a standard plant based medication. Just like silymarin there is need to discover further plant base sources to combat cancer and other diseases with no or less side effects. As preventing renal toxicity with use of inexpensive and useful drugs reduces hospitalization expenditure, also extenuate the rates of morbidity and mortality.

Based on the nephroprotective potential of related species and its polyphenolic compounds in animal models and in vivo protective actions of *A.* *hydaspica*. The present study was intended to examine the preventive/curative potential of the ethyl-acetate extract of *A. hydaspica* against CisPT-induced renal injuriousness and oxidative trauma in rats. We hypothesized that polyphenol-rich AHE would preclude CisPT-induced kidney damage due to its antioxidant action. In this study, we tried to find what influence AHE potentially had on the nephrotoxic and antineoplastic effects of CisPT treatment.

## Results

### Body and kidney weight

Considerable final body weight loss (*p* < 0.0001) was observed in CisPT-treated rats compared control group. On the contrary, a significant weight gain was noted in the CisPT + AHE and AHE + CisPT treated rats compared to CisPT treated rats. In AHE + CisPT treated group, body weight was similar to the control group. Moreover, there was a significant difference (*p* < 0.05) in kidney weight in CisPT + AHE and AHE + CisPT treated rats in contrast to the CisPT-treated rats (Table 1).

**Table 1:** Effect of Cisplatin AHE treatment on body and kidney weights of rats.

Group	Body weight (g)		Kidney weight (g)
	Initial	Final	
Control	218.3 ± 0.882	259.7 ± 0.667^b^	2.00 ± 0.03^b,^* *p* = 0.028
CisPT	220.0 ± 0.577	226.3 ± 0.66^a^	1.80 ± 0.11^a,^* *p* = 0.028
AHE alone	218.0 ± 0.577	258.3 ± 0.667^b^	2.00 ± 0.03^b,^* *p*=0.028
CisPT + AHE	220.3 ± 0.881	244.0 ± 0.577^a,b^	1.87 ± 0.03
AHE + CisPT	219.0 ± 0.576	254.7 ± 0.881^a,b,c^	1.99 ± 0.035^b,^* *p *= 0.03
CisPT + Sily	218.7 ± 0.667	253.7 ± 0.667^a,b^	1.985 ± 0.05^b,^* *p *= 0.03

Data expressed as mean ± SEM (*n* = 6).

*Sily* Silymarin.

Asterisks ^*^ indicates significant difference at *p* values mentioned in the table. Non-significant difference (*p* > 0.05) was recorded between control and AHE alone treated group in all parameters (One-way ANOVA followed by Tukey’s multiple comparison tests).

^a^Significant difference vs. control group at *p* < 0.0001.

^b^Significant difference vs. CisPT-treated group at *p* < 0.0001.

^c^Significant difference of AHE + CisPT pre-treated group vs. CisPT + AHE post-treated group at *p* < 0.0001.

### Effect of AHE on urine biomarkers

Effect of AHE counter to CisPT incited discrepancies in the kidney function markers in the urine were presented in Tables 2 and 3. Intensities of urinary markers of renal impairment viz urine specific gravity, RBCs, WBCs, and concentration of urea elevated considerably (*p* < 0.0001) while, urine PH was dropped significantly (*p* < 0.0001) in the CisPT treated group compared to control group. Inoculation of CisPT significantly (*p* < 0.0001) raised the level of urinary creatinine, albumin, and increase urinary protein excretion and reduced (*p* < 0.0001) the potency of creatinine clearance as compared to control group (Table 3). Administration of AHE before and after CisPT treatment markedly improved kidney function by significantly (*p* < 0.001) attenuating the above parameters. The urine profile of the AHE pretreatment group showed a non-significant difference in comparison to standard reference drug silymarin. Pretreatment of rats before CisPT injections significantly (*p* < 0.0001) restored the levels of urine biomarker enzymes in contrast to post-treatment indicating a protective effect.

**Table 2:** Effect of cisplatin (CisPT) and different treatments of AHE on urine profile.

Group		PH	Specific gravity	RBC/µl	WBC/µl	Urea (mg/dl)
Control		7.11 ± 0.05^b^	1.042 ± 0.011^b^	0.045 ± 0.011^b^	15.83 ± 0.29^b^	12.77 ± 0.589^b^
CisPT		5.6.033 ± 0.039^a^	1.489 ± 0.025^a^	13.12 ± 0.044^a^	76.53 ± 0.32^a^	41.07 ± 0.636^a^
AHE alone		7.107 ± 0.029^b^	1.041 ± 0.012^b^	0.046 ± 0.011^b^	15.77 ± 0.23^b^	12.57 ± 0.484^b^
CisPT + AHE		6.270 ± 0.05^a,b,^**^,d^	1.397 ± 0.033^a,b,d^	12.28 ± 0.038^a,b,d^	65.57 ± 0.34^a,b,d^	33.47 ± 0.48^a,b,d^
AHE + CisPT		7.037 ± 0.029^b,^ ^c^	1.076 ± 0.02^b,^ ^c^	0.626 ± 0.023^a,b,^ ^c^	16.10 ± 0.31^b,c^	14.67 ± 0.405^b,c^
CisPT + Sily		7.083 ± 0.032^b^	1.05 ± 0.015^b^	0.61 ± 0.012^a,b^	16.03 ± 0.32^b^	14.40 ± 0.305^b^

Values expressed as mean ± SEM.

*,**Significant difference at *p* < 0.022 and *p* = 0.0017 respectively. Non-significant difference (*p* > 0.05) was recorded between control and AHE alone treated group in all parameters (One-way ANOVA followed by Tukey’s multiple comparison tests).

^a^Significance at *p* < 0.0001 vs. control group.

^b^Significance at *p* < 0.0001 vs. cisplatin (CisPT) group.

^c^Significance at *p* < 0.0001 of AHE + CisPT pre-treated group vs. CisPT + AHE post-treated group.

^d^Significance at *p* < 0.0001 of CP + AHE treatment groups vs. CisPT + Sily group.

**Table 3:** Effect of cisplatin (CisPT) and different treatments of AHE on urine creatinine, creatinine clearance, albumin, and urinary proteins.

Group	Creatinine (mg/dl)	Creatinine clearance (ml/min)	Albumin (mg/dl)	Urinary protein (mg/dl)
Control	0.403 ± 0.055^b^	0.825 ± 0.064^b,^**	7.34 ± 0.171^b^	23.29 ± 0.76^b^
CisPT	1.407 ± 0.023^a^	0.409 ± 0.062^a,^** ^*p*=0.0013^	15.9 ± 0.458^a^	53.91 ± 0.482^a^
AHE alone	0.401 ± 0.055^b^	0.826 ± 0.065^b,^** ^*p*=0.0021^	7.28 ± 0.140^b^	23.29 ± 0.025^b^
CisPT + AHE	1.130 ± 0.091^a,b,^*^,d^ ^*p*=0.0228^	0.533 ± 0.059	13.7 ± 0.321^a,b^** ^*p*=0.0017,d^	45.72 ± 0.434^a,b,d^
AHE + CisPT	0.511 ± 0.038^b,c^	0.760 ± 0.079^b,^* ^*p*=0.0226^	8.87 ± 0.333^a,^* ^*p*=0.022,b,c^	27.17 ± 0.029^a,b,c^
CisPT + Sily	0.501 ± 0.039^b^	0.756 ± 0.077^b,^*	8.80 ± 0.20^a,^* ^*p*=0.0226,b^	27.82 ± 0.298^a,b^

Values expressed as mean ± SEM.

*,**Significant difference at *p* < 0.026 and non-significant difference (*p* > 0.05) was recorded between control and AHE alone treated group in all parameters (One-way ANOVA followed by Tukey’s multiple comparison tests).

^a^Significance at *p* < 0.0001 vs. control group.

^b^Significance at *p* < 0.0001 vs. Cisplatin (CisPT) group.

^c^Significance at *p* < 0.0001 of AHE + CisPT pre-treated group vs. CisPT + AHE post-treated group.

^d^Significance at *p* < 0.0001 of CisPT + AHE treatment groups vs CisPT + Sily group.

### Effect of AHE on renal function biomarkers

The serum profile indicates proper kidney function. Urea, BUN, serum creatinine, and uric acid levels determine the glomerular filtration rate and were considered as functional nephrotoxicity indices. CisPT induced nephrotoxicity was apparent from the elevated (*p* < 0.0001) levels of uric acid, creatinine, nitrite content, BUN, and serum urea, while serum albumin, protein, and globulin were considerable (*p* < 0.0001) decreased in contrast to control group (Table 4). Both pre- and post-treatment groups ameliorated the toxic effect of CisPT by markedly (*p* < 0.001) improving the serum urea, uric acid, creatinine, BUN, nitrite content, albumin, protein and globulin profile. However, substantial (*p* < 0.0001) restoration in the levels of the above-mentioned parameters was noticed in AHE pre-treated rats as compared to post-treated rats. AHE alone treatment group showed a non-significant difference in serum parameters in comparison with the control group corroborating with the non-toxic effect of the selected dose.

**Table 4:** Effect of cisplatin (CisPT) and different treatments of AHE on serum markers of kidney function.

Group	Serum creatinine (mg/dl)	Urea (mg/dl)	Uric acid (mg/dl)	Serum proteins (mg/dl)	Albumin (mg/dl)	Globulin (mg/dl)	BUN (mg/dl)	Serum nitrite (µM/ml)
Control	19.25 ± 0.43^b^	24.03 ± 0.55^b^	0.407 ± 0.0426^b^	78.06 ± 0.36^b^	19.7 ± 0.89^b^	58.36 ± 1.25^b^	9.167 ± 0.15^b^	14.5 ± 0.76^b^
CisPT	78.06 ± 0.36^a^	69.13 ± 0.47^a^	0.8067 ± 0.023^a^	39.25 ± 0.43^a^	9.6 ± 0.80^a^	29.65 ± 0.623^a^	23.13 ± 0.47^a^	81.67 ± 0.88^a^
AHE alone	19.06 ± 0.55^b^	30.03 ± 0.55^b^	0.4033 ± 0.044^b^	79.06 ± 0.55^b^	19.93 ± 0.64^b^	55.52 ± 0.486^b^	20.17 ± 0.44^b^	14.2 ± 0.61^b^
CisPT + AHE	55.72 ± 0.43^a,b,d^	62.60 ± 1.56^a,b,^**^,d^	0.76 ± 0.021^a,d,^**	45.72 ± 0.43^a,b,d^	12.53 ± 0.26^a,b,^*^,d^	33.18 ± 0.67^a,d^	13.03 ± 0.58^a,b,^**^,d^	43.17 ± 0.83^a,b,d^
AHE + CisPT	23.32 ± 0.44^a,b,c^	30.27 ± 0.73^a,^**^,b,c^	0.48 ± 0.038^b,c,^**	73.32 ± 0.44^a,b,c^	17.8 ± 0.153^b,c^	59.12 ± 1.08^b,c^	9.133 ± 0.17^a,b,c^	20.3 ± 0.91^a,^**^,b,c^
CisPT + Sily	23.97 ± 0.29^a,b^	23.73 ± 0.86^a,^**^,b^	0.477 ± 0.041^b^	73.97 ± 0.29^a,b^	17.93 ± 0.26^b^	56.04 ± 0.092^b^	12.87 ± 0.32^a,b^	20.73 ± 1.2^a,^**^,b^

Values expressed as mean ± SEM.

*,**Significant difference at *p* < 0.05 and p < 0.001 respectively. Non-significant difference (*p* > 0.05) was recorded between control and AHE alone treated group in all parameters (One-way ANOVA followed by Tukey’s multiple comparison tests).

^a^Significance at *p* < 0.0001 vs. control group.

^b^Significance at *p* < 0.0001 vs. Cisplatin (CisPT) group.

^c^Significance at *p* < 0.0001 of AHE + CisPT pre-treated group vs. CisPT + AHE post-treated group.

^d^Significance at *p* < 0.0001 of CisPT + AHE treatment groups vs. CisPT + Sily group.

### The protective action of AHE on renal antioxidant enzymes

Tables 5 and 6 exhibited the changes in the antioxidant enzymes in the various treatment groups. Antioxidant enzymes are repressed by CisPT and renal actions of superoxide dismutase, glutathione, and catalase are extensively attenuated. Single intraperitoneal (i.p.) intervention of CisPT induced a noteworthy (*p* < 0.0001) deterioration in SOD, QR, CAT, and POD enzyme profile. AHE significantly (*p* < 0.0001) recovered the activity level of these enzymes in post-treated and pre-treated rats in comparison to CisPT alone treated rats. Pretreatment with AHE showed marked elevation of POD (*p* < 0.05), SOD, CAT, and QR (*p* < 0.0001) as compared to AHE post-treatment, specifying that the presence of AHE before the CisPT intoxication provided better protection in contrast to its usage after the injury. The level of activity of SOD in the pretreatment group restored to the extent of the control group and non-significant difference in the above-mentioned parameters was recorded with standard treatment (silymarin). The oral dose of AHE alone showed no statistical variance in comparison to control group Table 5.

**Table 5:** Effect of cisplatin (CisPT) and different treatments of AHE on phase I antioxidant enzymes.

Group	POD (U/min)	SOD (U/mg protein)	CAT (U/min)	QR (nM/min/mg protein)
Control	10.85 ± 0.15^b^	1.752 ± 0.059^b^	35.5 ± 0.289^b^	93.49 ± 0.526^b^
CisPT	6.1 ± 0.058^a^	0.4069 ± 0.024^a^	17.45 ± 0.252^a^	58.25 ± 0.749^a^
AHE alone	11.3 ± 0.185^b^	1.778 ± 0.066^b^	35.93 ± 0.479^b^	93.84 ± 0.14^b^
CisPT + AHE	9.01 ± 0.115^a,b,d,^** ^*p*=0.0017^	1.026 ± 0.04^a,b,d^	21.93 ± 0.173^a,b,d^	65.94 ± 0.677^a,b,d^
AHE + CisPT	9.7 ± 0.058^a,b,c,^* ^*p*=0.021^	1.572 ± 0.055^b,c^	31.78 ± 0.129^a,b,c^	85.78 ± 0.229^a,b,c^
CisPT + Sily	9.89 ± 0.055^a,b^	1.584 ± 0.06^b^	30.93 ± 0.35^a,b^	85.98 ± 0.53^a,b^

Values expressed as mean ± SEM.

*,**Significant difference at *p* values mentioned within the table. Non-significant difference (*p* > 0.05) was recorded between control and AHE alone treated group in all parameters (One-way ANOVA followed by Tukey’s multiple comparison tests).

^a^Significance at *p* < 0.0001 vs. control group.

^b^Significance at *p* < 0.0001 vs. Cisplatin (CisPT) group.

^c^Significance at *p* < 0.0001 of AHE + CisPT pre-treated group vs. CisPT + AHE post-treated group.

^d^Significance at *p* < 0.0001 of CisPT + AHE treatment groups vs. CisPT + Sily group.

**Table 6:** Effect of cisplatin (CisPT) and different treatments of AHE on phase II antioxidant enzymes.

Group	GSH (µM/g tissue)	GR (nM/min/mg protein)	GST (nM/min/mg protein)	γ-GT (nM/min/mg Protein)	GPx (nM/min/mg Protein)
Control	17.41 ± 0.53^b^	150.4 ± 0.73^b^	139.3 ± 0.35^b^	394.4 ± 1.25^b^	129.7 ± 1.3^b^
CisPT	10.01 ± 0.33^a^	98.52 ± 0.82^a^	97.29 ± 0.64^a^	106.0 ± 1.17^a^	71.88 ± 0.825^a^
AHE alone	18.38 ± 0.67^b^	150.6 ± 0.41^b^	139.5 ± 0.44^b^	394.8 ± 1.38^b^	130.1 ± 1.10^b^
CisPT + AHE	13.24 ± 0.62^a,^**^,b,^** ^*p*=0.0013,d,^* ^*p*=0.022^	120.7 ± 2.3^a,b,d^	116.6 ± 0.61^a,b,d^	217.3 ± 1.36^a,b,d^	85.80 ± 1.23^a,b,d^
AHE + CisPT	16.01 ± 0.58^b,c,^* ^*p*=0.028^	140.4 ± 0.38^a,b,c^	132.7 ± 0.87^a,b,d^	367.4 ± 0.98^a,b,c^	120.7 ± 1.06^a,^** ^*p*=0.0017,b,c^
CisPT + Sily	16.12 ± 0.27^b^	139.1 ± 1.48^a,b^	132.5 ± 1.03^a,b^	366.5 ± 1.38^a,b^	120.3 ± 1.31^a,b^

Values expressed as mean ± SEM.

*,**Significant difference at *p* values mentioned within the table. Non-significant difference (*p* > 0.05) was recorded between control and AHE alone treated group in all parameters (One-way ANOVA followed by Tukey’s multiple comparison tests).

^a^Significance at *p* < 0.0001 vs. control group.

^b^Significance at *p* < 0.0001 vs. Cisplatin (CisPT) group.

^c^Significance at *p* < 0.0001 of AHE + CisPT pre-treated group vs. CisPT + AHE post-treated group.

^d^Significance at *p* < 0.0001 of CisPT + AHE treatment groups vs. CisPT + Sily group.

Furthermore, CisPT-treated rats showed significant (*p* < 0.0001) decrease in phase II antioxidant enzymes in renal tissue viz., glutathione (GSH), GR, GST, γ-GT, and GPx in comparison with the control group. The presence of AHE before or after CisPT intoxication significantly (*p* < 0.001) enhanced the activities of antioxidant enzymes as compared to CisPT-treated rats. The AHE pretreatment resulted in significant (*p* < 0.0001) restoration and normalization of above mentioned antioxidant enzymes as compared to AHE post-treatment group. Glutathione depletion is one of the main causes of CisPT induced nephrotoxicity and present results showed that AHE pretreatment restored the GSH to normal control values Table 6.

### Protective effect of AHE on oxidative trauma and inflammatory biomarkers

Table 7 reveals oxidative stress and inflammatory biomarkers in various treatment groups. CisPT persuaded oxidative stress was evident by markedly (*p* < 0.0001) increased levels of H_2_O_2_, nitrite, and MDA products in the tissues of rats that receive only CisPT in contrast to the control group. Alterations in tissue soluble protein, H_2_O_2_, nitrite content, and lipid peroxidation product (MDA) formation were significantly (*p* < 0.0001) attenuated by AHE post and pretreatment groups as compared to CisPT alone treated group. Pretreatment seems to be more pronounced in ameliorating the oxidative stress marker levels in contrast to post-treatment. Moreover, the pretreatment group showed similar effects to the standard drug silymarin-treated group. Renal NF-kB and IL6 levels as determined by the ELISA method are shown in Table 7. Renal tissue NF-κB and IL-6 levels of the rats in the CisPT group are significantly elevated relative to those of the control group. However, AHE pretreatment significantly ameliorated the activity of transcription factor NF-κB and returned pro-inflammatory cytokine IL-6 levels to values near those of control. AHE pretreatment showed significant preventive potential compared to its administration after CisPT. These findings point out the potential of AHE to recover or stabilize the renal functions through an increase in the antioxidant influence, diminishing lipid peroxidation, and inflammatory biomarkers via quenching the free radicals, besides cumulating intracellular concentration of phase I and phase II antioxidant enzymes.

**Table 7:** Effect of cisplatin (CisPT) and different treatments of AHE on kidney tissue protein and oxidative stress and inflammatory biomarkers.

Group	Protein (µg/mg Tissue)	H_2_O_2_ (nM/min/mg Tissue)	Nitrite (µM/ml)	MDA (nM/min/mg protein)	NF-κB (ng/ml)	IL-6 (pg/ml)
Control	2.53 ± 0.056^b^	1.743 ± 0.038^b^	27.84 ± 1.167^b^	5.026 ± 0.301^b^	8.11 ± 1.18^b^	21.52 ± 0.86^b^
CisPT	0.565 ± 0.176^a^	4.366 ± 0.029^a^	86.41 ± 1.381^a^	11.19 ± 0.659^a^	98.20 ± 2.03^a^	121.67 ± 1.13^a^
AHE alone	2.47 ± 0.019^b^	1.707 ± 0.013^b^	25.46 ± 1.302^b^	5.01 ± 0.1832^b^	8.01 ± 1.01^b^	19.13 ± 0.71b
CisPT + AHE	1.425 ± 0.114^a,b,d^	3.478 ± 0.026^a,b,d^	50.67 ± 1.180^a,b,d^	9.40 ± 0.7349^a,d,^* ^*p*=0.022^	50.27 ± 1.91^a,b,d^	62.89 ± 1.02^a,b,d^
AHE + CisPT	2.183 ± 0.063^b,c,^** ^*p*=0.0016^	1.968 ± 0.048^a,^* ^*p*=0.028,b,c^	30.07 ± 1.589^b,c^	6.86 ± 0.2638^b,c,^*^p=0.026^	13.25 ± 0.69^b,c^	30.10 ± 0.88^b,c^
CisPT + Sily	2.199 ± 0.052^b^	1.996 ± 0.07^a,^* ^*p*=0.022,b^	29.74 ± 1.272^b^	6.22 ± 0.2167^b^	13.06 ± 0.71^b^	29.01 ± 0.90^b^

Values expressed as mean ± SEM.

*,**Significant difference at *p* values mention in the table. Non-significant difference (*p* > 0.05) was recorded between control and AHE alone treated group in all parameters (One-way ANOVA followed by Tukey’s multiple comparison tests).

^a^Significance at *p* < 0.0001 vs. control group.

^b^Significance at *p* < 0.0001 vs. Cisplatin (CisPT) group.

^c^Significance at *p* < 0.0001 of AHE + CisPT pre-treated group vs. CisPT + AHE post-treated group.

^d^Significance at *p* < 0.0001 of CisPT + AHE treatment groups vs CisPT + Sily group.

### Effect of AHE treatment on protein expression of IL-6 and NFкB p65 signal transducers

**Figure 1:**
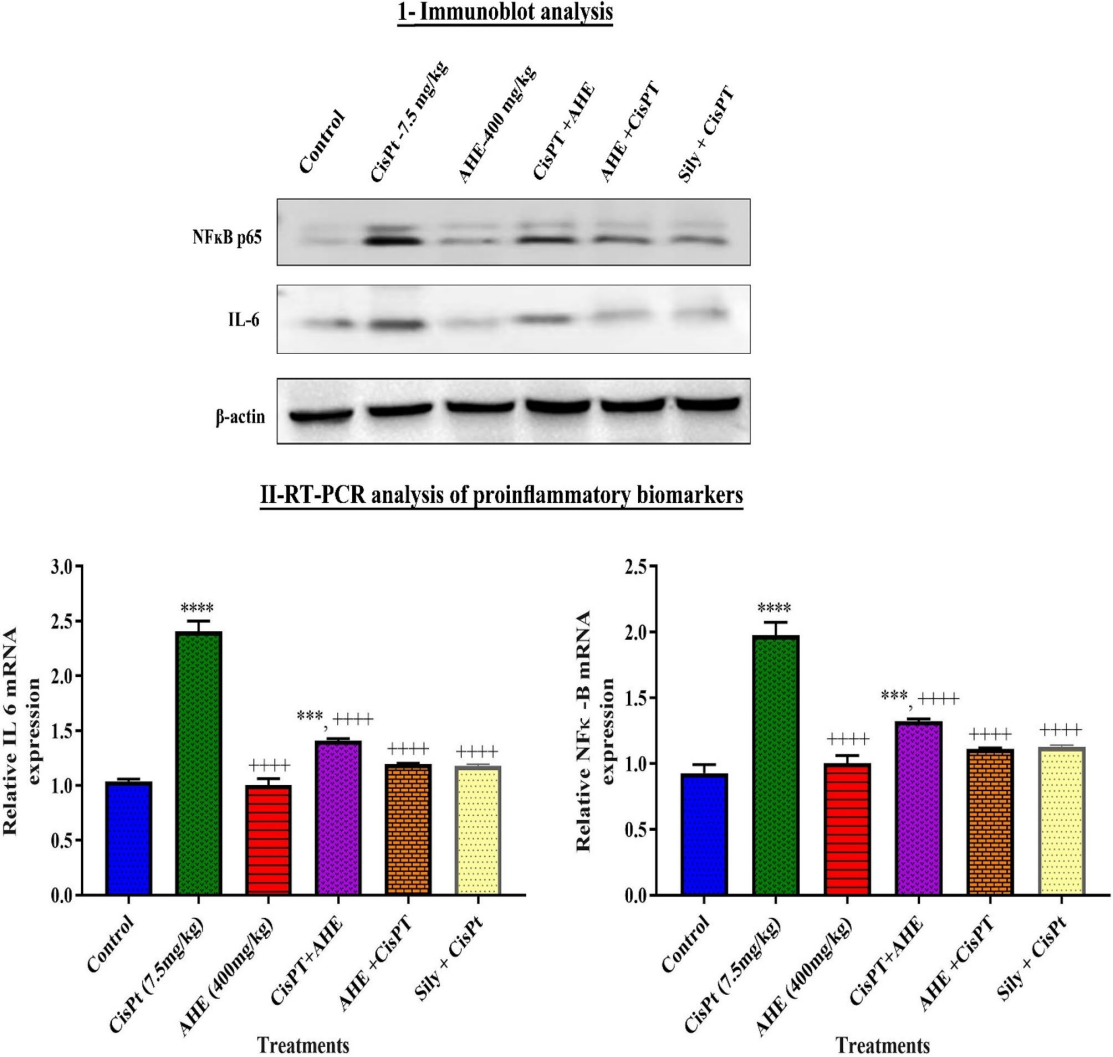
I-Immunoblot analysis of rat kidney tissues showing the expression of NFĸB and IL-6 protein expression. β-actin was used as a protein loading control. II-Quantification of mRNA expression of inflammatory mediators (IL-6 and NF-κB) in kidney tissue. Asterisk ***, **** represent significance at *p* < 0.001 and *p* < 0.0001 from control group. Non-significant difference (*p* > 0.05) was recorded between control and AHE alone treated group in all parameters (One-way ANOVA followed by Tukey’s multiple comparison tests analyzed by Graph pad prism 5 software https://www.graphpad.com/download/).

Organ toxicities induced by CisPT were categorized by a significant increase in kidney tissue protein expression of IL-6 and NF-κB p65 of the CisPT group when compared with the control group. Furthermore, immunoblot analysis demonstrated that AHE + CisPT treated group significantly downregulated kidney tissue protein expression of IL-6 and NF-κB p65 (Fig. 1I) when compared with CisPT alone treated group. The effect of the AHE + CisPT treatment group is similar to Sily + CisPT group.

### Quantification of mRNA expression of inflammatory mediators (IL-6 and NF-κB) in kidney tissue

Our results revealed that there is a significant increase in the mRNA expression of inflammatory markers; IL-6, and NF-κB expression in the kidney of CisPT-treated animals as compared to the control group (Fig. 1II). These responses were markedly attenuated by pretreatment with AHE when compared with CisPT (7.5 mg/kg) treated group in kidney tissues.

### Histopathology

**Figure 2:**
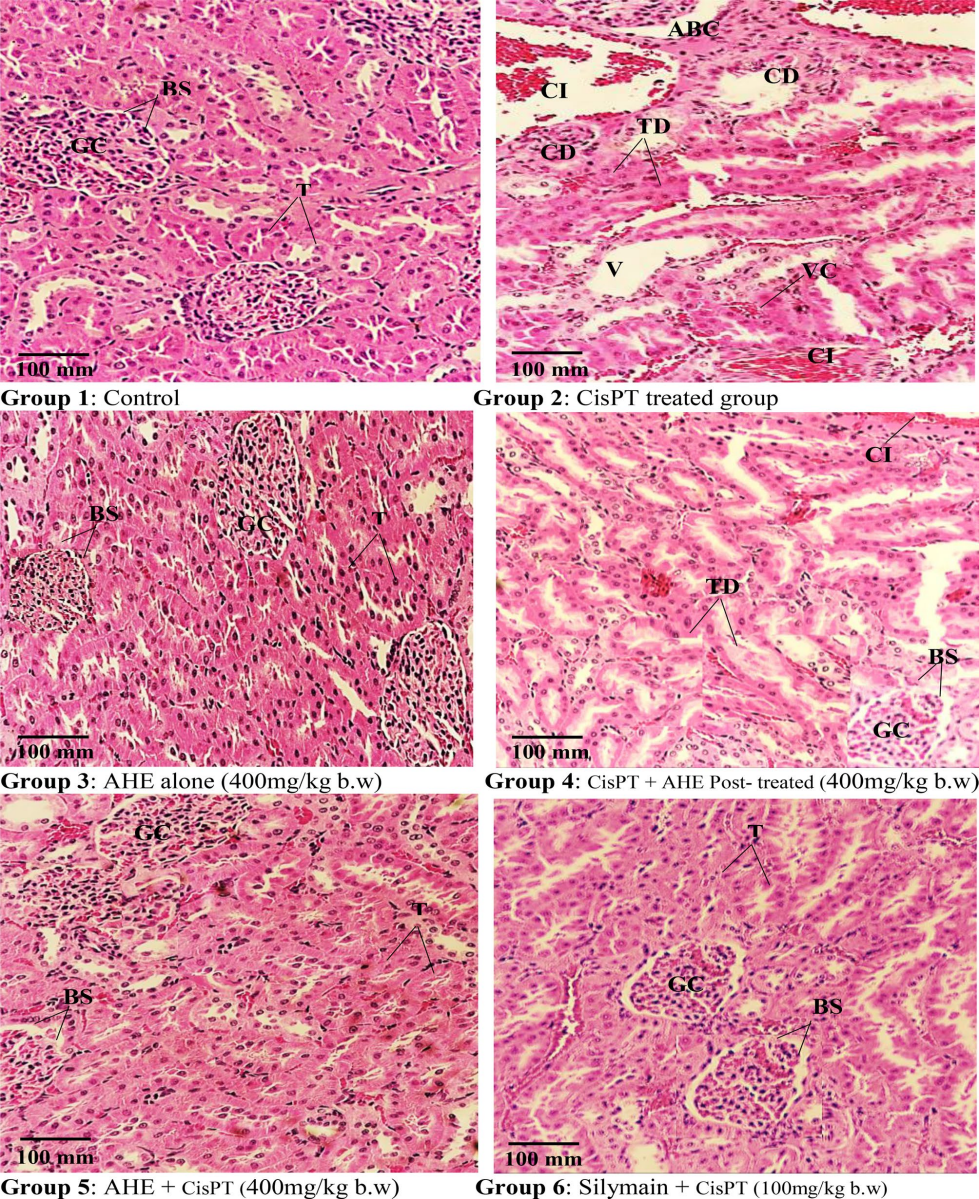
Histopathological effect of Cisplatin and protective effect of AHE in rat kidney (H&E staining, magnification ×40). Group 1: Renal section from control rats showing normal kidney morphology. Group 2: renal sections from CisPT-treated rats reveal degenerative changes, atrophy, capsule distortion, and inflammatory cells. Group 3: Represents renal section from AHE alone treated rats. Group 4: AHE post-treatment showed tubular dilation and cellular infiltrations. Group 5: AHE Pre-treatment results in significant protection against CisPT induced renal injury. Group 6: Showed the protective effect of Silymarin treatment. *AHE* *A. hydaspica* ethyl acetate fraction, *CisPT* Cisplatin, *GC* Glomerular capsule, *BS* Bowman’s space, *T* Tubules, *CI* Cellular infiltrations, *ABS* alteration in Bowman’s space, *TD* Tubule dilation, *CD* Capsule distortion, *VC* vascular congestions, *V* vacuolization.

Renal histopathological alterations in different treatment groups are shown in Fig. 2 and Supplementary File S2 Normal histology of rat kidneys (glomeruli structure encapsulated in Bowman’s capsule, tubules, interstitium, and blood vessels) was apparent in the control and AHE alone treated groups. After inoculation of a single dose of CisPT (7.5 mg/kg, i.p.), the CisPT-treated group exhibited severe renal damages, tubular necrosis, remarkable vacuolization, dilatation of Bowman’s capsule, glomerular atrophy, the disintegration of the tubular epithelium, pyknotic nuclei, proteinaceous casts in renal tubules, deterioration, necrosis, and detachment of the proximal tubular epithelial cell lining, shedding of the apical microvilli and lost cellular details. The kidney of rats treated with AHE (post and pretreatment) to CisPT exposed not as much of histological damage in renal corpuscles and renal tubules. Mild tubular degeneration with luminal dilatation was seen within the renal cortex of the post-treatment group, while pretreatment showed normal appearance of glomeruli and bowman’s capsule, only some of the glomeruli appear degenerated, the cellular structure appears normal with no proteinaceous cast observed.

Morphological studies make evident that the renal tubule system is the site of maximum cisplatin damage while a shielding influence of AHE was apparent in the tubule system. AHE post-treatment produced less pronounced effects and mildly dilated tubules were seen while pretreatment with AHE improved the explicit changes provoked by CisPT. Silymarin was used as a reference drug which showed protective effects against CisPT induced renal deteriorations. Silymarin pretreatment reversed the CisPT induced pathogenesis, relative to AHE pretreatment and control groups. The histopathological changes confirmed the biochemical findings.

### AHE does not attenuate the anticancer properties of CisPT

**Figure 3:**
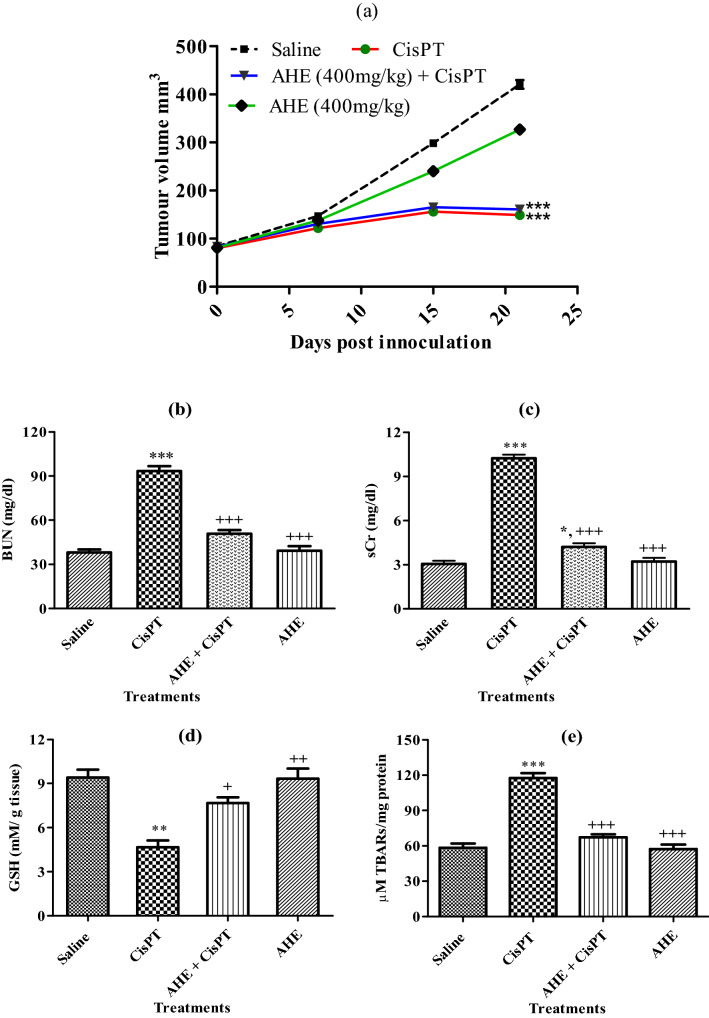
AHE does not attenuate the anticancer properties of CisPT in vivo mouse tumor model and concurrently confers nephron-protection. Subcutaneous HCT 116 tumors cell lines were inoculated in male BALB/c mice to induce tumors. Mice were then treated with Saline, CisPT, AHE + CisPT (**a**) Tumor size was measured once before CP administration and then weekly. Results are expressed as mean ± SEM, *n* = 8 animals per group. *** indicated *p* < 0.0001 significant difference of saline versus CisPT and AHE + CisPT groups tumor final volumes (**b)** BUN and (**c**) sCr, (**d**) GSH and (**e**) TBARs levels were assessed at the termination of the experiment. Esterics *, ** and *** indicated *p* < 0.05, *p* < 0.001 and *p* < 0.0001significance from saline group while + , ++ and +++ indicated *p* < 0.05, *p* < 0.001 and *p* < 0.0001significance from CisPT group results are expressed as mean ± SEM, *n* = 8 animals per group (One-way ANOVA followed by Tukey’s multiple comparison tests analyzed by Graph pad prism 5 software https://www.graphpad.com/download/).

It was of deep concern to check out if the usage of AHE compromises the antitumor efficacy of CisPT or not. Subcutaneous tumors were induced in male BALB/c mice using HCT 116 cell lines and tumor-bearing animals were treated with CisPT, AHE, or both. CisPT inoculation significantly inhibited tumor progression (Fig. 3a), and conclusively, AHE did not attenuate this antitumor response. Importantly, increased BUN (Fig. 3b), and sCr (Fig. 3c) levels were prevented when CisPT-inoculated tumor-bearing mice were co-treated with AHE. AHE also attenuated oxidative stress in the kidney, as evidenced by the significant increase in GSH (Fig. 3d) and reduction in lipid peroxidation (Fig. 3e). Because the anticancer activity of CisPT was unaffected, it can also be concluded that the concurrent nephroprotection is not due to the sequestration of CisPT by AHE in the blood. Therefore, AHE does not interfere with the antitumor properties of CisPT, while concurrently defending the renal toxicity induced by CisPT.

## Discussion

Acute renal damage is accompanying with enhanced oxidative trauma, and several endogenous and non-natural antioxidants that diminish oxidants are valuable in cell-based and animal trials. In the current trial, we investigated the potential protective effect of AHE on cisplatin-induced nephrotoxicity using rodent models. Drug-induced deteriorations on kidney function and tubular lesions can be easily evaluated by using a rodent model, as their intra‐renal enzyme dissemination is more or less analogous to humans^27^. After a single-dose of CisPT, significant concentrations of the drug were accumulated in the kidney for as long as 1 month or even 3 months^28^. Multiple mechanisms which are involved in CisPT-induced renal damage shared nearly common modulator. Oxidative stress seems to be a common trigger in most cases. Urine analysis is a direct indicator of kidney function and acid–base balance. The elevated level of urine creatinine, urea, protein, and albumin and decrease creatinine clearance are general markers of drug-induced kidney injury^29^. We observed abnormal proteinuria and hematuria in the CisPT inoculated groups, indicative of nephrotoxicity. Urine specific gravity associated with urine osmolality provides critical details of hydration status. CisPT altered urine specific gravity and pH. Treatment with AHE before CisPT injection showed pronounced improvement in the above-mentioned parameters. The protective effect of AHE might be linked to the synergistic effect of active metabolites present in the extract. Our findings showed consistency with Ahn and colleagues describing the protective effect of pretreatment with green tea polyphenols against CisPT induced changes in Kidney functions^25^. We investigated different pharmacological activities of AHE in our previous investigations and identified different active metabolites. Supplementary File S3; Table 2 revealed the bioactive metabolites of AHE.

CisPT inoculation to rats may induce a drop in glomerular filtration rate, accompanied by increased serum creatinine, BUN, urea, and uric acid. These findings are in correspondence with earlier outcomes in different studies revealing that a high concentration of tissue injury biomarkers in serum might be due to the renal tissue impairment, tubular blockade, and/or the back-leakage of the kidney tubules^30^. Additionally, CisPT administration decrease kidney tissue protein content and AHE treatment result in significant restoration of kidney tissue protein content. These functional instabilities in CisPT exposed rats point toward the ability of this drug to prevent protein synthesis in the tubular cells or to recruit lipid peroxidation and free radical generation in nephrons^31–33^. On the other hand, the treatment of AHE before or after CisPT administration considerably improved the kidney function biomarkers in serum, however, pretreatment showed a more pronounced protective effect compared to post drug administration. The synergistic action of secondary metabolites might be responsible for the actions of AHE. Our outcomes are consistent with prior studies signifying the preventive and curative potential of Bern date extract by ameliorating the kidney functions. The nephroprotective ability attributed to the presence of antioxidant activity of polyphenolics in Berne dates^34^.

The current examination revealed that AHE not only ameliorated CisPT induced alterations in serum kidney function biomarkers, however, but it also executed affirmative influence on rat’s body and kidney weights. Analogous findings were stated by Moneim et al.^50^ who found that the methanolic leaf extract of *Azadirachta indica* showed protective action contrary to CisPT-induced kidney damage in rats, where antioxidant constituents in the extract were proposed to be the source of protection^35^.

Our investigation revealed that CisPT induced redox disproportion, as verified by an upsurge in MDA and NO activity, reduced GSH intensity, and diminished expression of POD, SOD, CAT, GPx, γ-GT, GST, and GR in renal tissue. Oxidative stress has been proposed to be the preliminary mechanism of CisPT-triggered nephron damage. The obvious decline in renal GSH expression in reaction to CisPT inoculation was due to its depletion by the quenching of free radicals. The depletion of the GSH pool may be due to the accumulation of MDA, and compromised ability of kidney to filter harmful H_2_O_2_, and lipid peroxides. Such findings were also noticed by other researchers^30^. The enzymatic antioxidant enzymes govern central function in the removal of reactive oxygen and nitrogen products which are predisposed for cellular oxidative injury^36^. AHE treatment was able to significantly restored antioxidant enzyme activities in the kidney tissues. Reno-protective actions of AHE against CisPT induced nephrotoxicity may be due to its chemical composition as AHE fraction of *A. hydaspica* is opulent in flavonoids and phenolic. Flavonoids compounds possess antioxidant and anti-inflammatory actions by quenching free radicals and impeding oxidative reactions of lipids^37^. The accurate pathway whereby ROS are created in AKI is not clear, declining ROS production and oxidative harm are impending therapeutic goals. Antioxidants can interfere in the initial stage of pathogenesis by conclusively abolishing oxidative product origin. Similarly, polyphenol-rich green tea revealed a marked reduction in the CisPT-induced ROS via an increase in renal SOD and CAT level^38^. We noticed an upsurge in renal MDA, NO, and H_2_O_2_ levels in CisPT group. Our findings ratify preceding studies, which demonstrated an increase in lipid peroxidation and subsidence of the antioxidant defense system in the kidney after CisPT treatment^30, 35^. Results revealed that AHE could prevent the renal injury induced by CisPT and did not affect the anticancer potential of CisPT, these findings evidence the imperative function of AHE in blocking drug-prompted nephrotoxicity. Likewise Abdel Moneim et al. testified that *A. indica* may limit lipid peroxidation by extinguishing free radicals and amplifying intracellular absorption of glutathione due to the amount of flavonoids^35^. Initiation of renal injury by chemotherapeutic drugs is expected to be instantaneous response including sensitive proteins in the renal tubules. Hence, the preventive agent must be prevailing in nephrons before the damaging impact of drugs or should be administered simultaneously with the chemotherapeutic drug to keep away its side effects. These effects are in agreement with the prior research of Azu et al*.* describing the potential of Kigelia Africana fruit extract (KAFE) against CisPT persuaded nephrotoxicity^39^. We detected in a previous finding that AHE is rich in catechins and 7-*O*-galloyl catechin. The prevention of CisPT induced oxidative stress might attribute to the presence of polyphenols. Previous studies provide evidence that catechins prevent drug-induced lipid peroxidation and ameliorated kidney damage due to their antioxidant potential^40^.

In our previous investigations we observed an increase in the secretion of TNF-*α* and IL-6 in reaction to CisPT inoculation, indicating that CisPT triggered inflammation in the testis. A great body of evidence proposed that CisPT generates oxidative trauma, leading to inflammation, which proceeds the amplified formation of TNF-*α* and IL-6^23^. The rise in TNF-*α* is linked to the surplus generation of ROS, various cytokines, and cell cycle regulatory elements that trigger several downstream signaling pathways including nuclear factor kappa B (NF-*κ*B) regulated stimulation of various target genes^34^. NF-κB is a pro-inflammatory transcription factor that controls the expression of lots of proteins intricate in inflammation, including cytokine IL-6. In the kidneys, IL-6 production is persuaded within a few hours of the inception of acute renal damage, and production persists to be augmented for several days. Thus, IL-6 might play as the protagonist in the pathogenesis of kidney ailment^41^. It has been observed that CisPT-induced renal tubular necrosis boosts inflammatory activities and stimulates NF-κB and IL-6 activity at the site of tissue damage. Activation of NF-κB in response to CisPT-induced oxidative stress plays a critical part in the renal inflammatory responses by regulating gene expressions of cytokines, adhesion molecules, and growth factors^42^. Our data revealed that cisplatin administration considerably inclined the level of expression of IL6, and NF-κB p65 as comprehended in the calorimetric and immunoblot and analysis in the kidney tissue. Also, RT-PCR analysis revealed an elevation in the expression of the IL-6 and NF-κB p65. Administration of AHE before CisPT considerably lowered the expression of IL-6 and NF-κB p65 in the kidney tissue by providing antioxidant defense against CisPT deteriorations reflecting the anti-inflammatory efficacy of AHE. AHE treated groups did not show any NF-κB and IL-6 over-activation, which further endorses its protective role through inhibition of inflammation and enhancement of antioxidant enzymes due to the occurrence of various secondary metabolites including catechins. Catechins are protective against LPS induced renal injury via anti-apoptotic, anti-inflammatory, and antioxidant mechanisms^26^. The examination of renal histo-architecture is necessary to validate the biochemical findings. Glomerular capillary tufts size contraction, Bowman’s capsule dilation, deterioration, necrosis, and detachment of the epithelial cell lining of proximal tubules, flaking of the apical microvilli and various discharges within the distended tubular Lumina of distal and proximal convoluted tubules were seen at the cortical-medullary area in the kidney sections of the CisPT inoculated rats. On average, the tubules were entirely collapsed and cellular intricacies have vanished. Analogous histopathological findings were demonstrated previously in the kidney of rats treated with altered doses of CisPT^30, 43–45^. Histopathological impairments may affect the renal tubules absorption efficacy and compromise the nephron functions that outcomes in kidney malfunction^46^. AHE treatment significantly ameliorates the damaging effects of CisPT on renal morphology. Phenolic constituents, principally flavonoids, are potent sources of antioxidants, and several plants contain flavonoids, and these constituents employ defensive influence against CisPT-prompted nephrotoxicity^47^. Previous research approved that silymarin possesses positive effects on preventing or decreasing the severity of CisPT nephrotoxicity, and regarded as a potentially pragmatic candidate in combined chemotherapy regimens by functioning as a potent quencher of radical species in the kidney, thus, precluding the toxic influence of CisPT at both histological and biochemical levels, AHE might work similarly as silymarin. Silymarin has a mixture of three flavonolignans that can scavenge free radicals and increase GSH levels^48^.

In an attempt to restrain adverse secondary effects of CisPT without compromising its anticancer efficiency, we examined whether AHE which possesses bioactive metabolites of potential therapeutic significance can supplement the anti-tumor activity of CisPT in a xenograft mouse model and the potential protective ability of AHE versus CisPT-prompted renal injury. In the tumor-bearing mice, substantially enlarged kidneys masses were recorded in the CisPT-inoculated animals. However, significant amelioration of kidney weights was noticed in AHE co-treatment group. The high intensity of serum biomarkers of kidney damage i.e., BUN, and Cr was recorded in CisPT-injected animals, evidencing malfunctioned kidneys in tumor-bearing mice treated with CisPT. Co-treatment with AHE significantly prevented CisPT-triggered deterioration in renal function biomarkers. In rationality with the nephrotoxic trail on a rat model, AHE usage with CisPT significantly reduced intensity of oxidative stress markers in the tumor xenograft model. The tumor size was effectively reduced in CisPT + AHE co-administered animals indicating effective control of the rate of tumor growth by CisPT and suggesting that AHE did not interfere with the antitumor effectiveness of CisPT. Remarkably, AHE did not affect the anti-tumor activity of CisPT while preventing its adverse nephrotoxic perspective. This is potentially beneficial interaction. Combining AHE as a supplementary antioxidant therapy with CisPT based chemotherapy could be a vital stratagem that has the potential for refining the antineoplastic activity of CisPT. However, the exact mechanism of action of AHE should be further elucidated as it consists of multifarious metabolites; therefore, further isolation work is needed to elucidate anti-inflammatory and antioxidant actions.

### Limitations of the study

Additional investigations are necessary to endorse the current outcomes using molecular tools and immunohistochemical procedures. Hence, studying gene expression of GSH, oxidative stress biomarkers, and inflammatory biomarkers through RT-PCR and their protein expression by western blotting is suggested. Advanced testing to generate an empirical basis for the consumption of *A. hydaspica* as supplementary therapy with cisplatin in the clinic will be aimed, to elucidate the mechanism of anti-inflammatory and anti-apoptotic signaling, the reformative proficiency of the kidneys, and renal cellular transport. Furthermore, due to shortage of funding currently we are unable to study the mechanism of protection in the xenograft mice model. Therefore, investigating the detailed mechanism of protection and how *A. hydapica* preserve the anticancer potential of CisPT in tumor models will be the focus of further investigations.

## Conclusion

To date, this is the first scientific evidence signifying the protective effect of AHE on CisPT nephrotoxicity in rats. AHE protects against CisPT nephrotoxicity without affecting its anti-cancer properties. The results highlight that AHE may defend CisPT-induced renal toxicity by suppression of CisPT intervened deterioration in renal tubular cells of rats. AHE provided a more pronounced protective effect against cisplatin-induced nephrotoxicity when administered before CisPT dose. The mechanism of nephroprotective by AHE could be due to the antioxidant and free radical scavenging activities of AHE*.* The present results suggested that AHE might be a potential candidate in preventing CisPT nephrotoxic effects. However, until now, there is no single acceptable herbal agent that can prevent CisPT nephrotoxicity to be endorsed clinically.

## Methods

### Collection of plant and preparation of AHE extract

*Acacia hydaspica* (Aerial parts) were collected from Kirpa Charah area Islamabad, Pakistan. Pakistan. Dr. Sumaira Sahreen recognized the plant, and the specimen was deposited (Accession No. 0642531) in the Herbarium of Pakistan, Museum of Natural History, Islamabad. The detailed procedure of *A. hydaspica* methanol extract preparation and fractionation is described in our earlier report^49^, and AHE; the most potent fraction under our various in vitro and in vivo inspections^15, 49^ was chosen for current in vivo research. The use of plant in the present study complies with international, national and/or institutional guidelines. No specific consents were requisite for the collection of plants as the regions were not in reserved-ownership or secure in any way and the field area did not involve endangered or protected species. We didn’t collect more than the minimum number of specimens necessary for the accomplishment of our research.

### Dose selection and preparation

In our previous investigation, acute toxicity testing of AHE was monitored, and oral doses i.e., 100, 200, 400, 1000, 2000, 3000 and 4000 mg/kg body weight (BW) were tested following the recommendations of Organization for Economic Cooperation and Development (OECD) for testing of chemicals for acute oral toxicity^50^. Details of experiments were reported in our previous investigations^21, 51^. AHE was found to be safe at all tested doses (up to 4000 mg/kg BW) and it did not induce any noxious symptom in rats like sedation, convulsions, diarrhea, and irritation^22, 24^. The dose of plant extract and silymarin were selected based on our pilot experiment and previous lab testing confirm the efficacy of these selected doses^22, 24, 52^. CisPT injection was purchased from Sigma-Aldrich (St. Louis, MO, U.S.A.). CisPT dose (7.5 mg/kg bw) was chosen based on earlier literature to bring acute kidney damage^39^. Silymarin (100 mg/kg BW) and AHE (400 mg/kg BW) were dissolved in distilled water just before treating.

### Experimental design

Male Sprague Dawley rats (*n* = 36, 200–225 g) were obtained from primate facility (Quaid-i-Azam University, Islamabad). The rats were placed in steel cages under favorable conditions (12 h light/dark cycle at 25 ± 3 °C). Rats were fed with a standard pellet diet and tap water ad libitum. National institute of animal health (NIH) guidelines for the care and use of laboratory animals were strictly followed for experiments^53, 54^. The investigational procedure (Bch#264) was permitted by the ethical board of Quaid-i-Azam University. The study proposal was acquiescent to previous examinations^43, 55^ with minor amendments. The study is reported in accordance with ARRIVE guidelines^56^. Animals were separated into six groups (*n* = 6). After acclimatization to laboratory conditions, the rats were divided into groups given distilled water. The body weight of rats was measured before the onset of the experiment and at the end of the experimental procedure.

Control group: no treatment.

CisPT treated group: inoculated with 1 dose of CisPT (7.5 mg/kg BW, i.p.) only.

AHE treated group: orally received AHE (400 mg/kg BW) for a week.

CisPT + AHE group (Post-treated): given CisPT injection (7.5 mg/kg BW, i.p.) and AHE (400 mg/kg BW/day, p.o.) for 7 days^57^.

AHE + CisPT group (pre-treated): received 400 mg/kg BW/day, p.o. 7 days before CisPT (7.5 mg/kg BW, i.p.) injection and 7 days after the injection.

Silymarin + CisPT group: received 100 mg/kg BW, p.o for 7 days before CisPT (7.5 mg/kg BW, i.p.) and 7 days after the injection. The pretreatment groups were given the plant extract or silymarin 1 week before the Cisplatin injection which is counted as day 1, Further treatments were given for 7 days after the injection in order to evaluate the effect of plant administration before Cisplatin inoculations. For that purpose, the post administration was same for all groups.

One week after the end of treatments, urine was collected and stored at − 70 °C for further analysis. Animals were euthanized by CO_2_ inhalation as anesthesia. Blood was collected by cardiac puncture and submitted to centrifugation at 500 × *g* for 15 min at 4 °C to obtain serum. The serum was stored at − 80 °C for subsequent biochemical analysis. The kidneys were extracted and rinsed with ice-cold saline to remove debris, subsequently, the right kidney was preserved with liquid nitrogen and kept − 80 °C for enzymatic investigations whereas the left kidney was processed in 10% phosphate-buffered formalin for histology.

### Physical analysis of urine

White blood cells (WBCs) count, red blood cells (RBCs) count, specific gravity, and pH were analyzed from urine samples via commercial diagnostic kits (MediScreen Urine Strips, Orgenics, France).

### Biochemical examination of urine and serum

Albumin, urea, total protein, creatinine, and creatinine clearance in urine were measured by commercial diagnostic kits (MediScreen kit France). The estimation of bilirubin, total protein, and Blood urea nitrogen (BUN) in serum was done by using standard diagnostics kits of AMP (Krenngasse 12, 8010 Graz, Australia).

### Biochemical analysis of tissues

#### Homogenate preparation

100 mM KH_2_PO_4_ buffer having 1 mM EDTA, pH 7.4 was used to homogenize each renal tissue sample (100 mg). Then centrifuged (12,000*g* at 4 °C for 30 min), was carried out to eliminate cell debris. The supernatant was collected and stored in aliquots at − 20 °C for lipid peroxidation products, antioxidant enzymes, nitrite content, and H_2_O_2_ assays.

### Tissue protein quantification

The total soluble proteins were calculated by Lowry et al. technique^58^. Potassium phosphate buffer was used to homogenize tissue samples (100 mg). Then centrifugation (10,000*g* at 4 °C) for 15–20 min was carried for the supernatant. To 0.1 ml of supernatant, alkaline solution (1 ml) was added, vortexed, and followed by 30 min incubation. The absorbance change was calculated at 595 nm. To measure out the concentration of serum proteins in the sample, bovine serum albumin (BSA) standard calibration curve was utilized.

### Enzymatic antioxidant status

POD and CAT activity was calculated by the previously established procedure^59^. SOD activity was assessed by Afsar et al. method^22^. The Quinone reductase quantity in renal samples was calculated as cited formerly^60^. Reduced glutathione activity was checked as described by Jollow^61^. The design of Habig et al.^62^ was monitored for the estimation of GST potency. Carlberg and Mannervik's method was utilized to estimate Glutathione reductase activity in tissue samples^63^. Glutathione peroxidase activity was measured as described in another study^21^. Orlowski et al. scheme was used to check the action of γ-glutamyl transpeptidase^64^.

### Assessment of oxidative stress markers

The protocol of Iqbal et al.^65^ was adopted with slight modifications for the assessment of lipid peroxidation. Hydrogen peroxide quantity in renal samples was estimated by the scheme defined earlier^66^. Griess reagent was used for the execution of the nitrite test^67^.

### Assessment of inflammatory biomarkers

#### Calorimetric measurement

Kidney tissue homogenate was made with phosphate buffer saline (50 mM, pH 7.4) containing 1% protease inhibitor cocktail (Sigma-Aldrich Co, St Louis, MO, USA). The supernatant was pipetted out after centrifugation (4000*g* at 4 °C for 20 min), and the protein content was determined by the Lowery et al. protocol^58^. Measurement of NF-κB p 65 (CSB-E13148r) and IL-6 (CSB-E04640r) was done according to the commercial kits (CUSABIO Technology LLC) manual.

### Gene expression analysis of inflammatory biomarkers

Total RNA from the frozen kidney tissue of each rat was extracted by using a kit, according to the manufacturer's instructions (Promega Cat. # Z3101). The cDNA synthesis was performed using the Abi kit. The reaction mixture was prepared to contain 10 µl fast-start Universal SYBR Green Master (Roche, Germany), 6 µM reverse primers, and 10 µg cDNA, with RNAase free water added to a total volume of 20 µl. The amplification and real-time analysis were done for 40 cycles with the following factors; 95 °C (10 min) to activate of FastStart Taq DNA polymerase; 60 °C (1 min) for amplification and real-time analysis^68^. The gene expression levels were determined using 2^−ΔΔCT^. Primer sequences used are shown in Supplementary File S1; Table 1.

### Western blot analysis of inflammatory biomarkers

Briefly, rat kidney tissue samples were lysed in RIPA buffer supplemented with freshly added protease and phosphatase inhibitor cocktail 1:100 (Sigma), and protein concentration was determined by Bradford assay^69^. The previously developed procedures with slight modifications were used to perform SDS-PAGE and western blot investigations^70, 71^. For SDS-Page, equal amounts of proteins were loaded using BIO-RAD Mini protein TGX precast gels on the bio-rad Mini protein tetra system (10025025 REVA). After protein separation by gel electrophoresis, the proteins were transferred to PVD membranes using a bio-rad trans blot Turbo transfer system. PVD membranes (bio-rad) were blocked for 2 h with either 5% BSA (Sigma) or 5% nonfat dry milk (Bio-Rad, Cat. #170-6404) then incubated with primary antibody overnight at 4 °C. The following antibodies were used: IL-6 (ab9324), NFκB p65 (ThermoFisher Scientific Catalog # 2A12A7), and β-actin (Abcam Cat. # ab49900). The antibodies signals were detected by ECL western blotting substrate (bio-rad) and blots were visualized using the bio-rad Gel documentation system (ChemiDoc MP System).

### Histopathological examination

For histopathological examination, renal tissues were stabilized in a fixative having absolute 85 ml of alcohol, 5 ml of glacial acetic acid, and 10 ml of formaldehyde (40%). After dehydration steps, tissue samples were mounted in paraffin to make units for microtomy. Tissues were sectioned 4–5 µm with a microtome and stained with Hematoxylin–Eosin (H&E) and studied below a light microscope (DIALUX 20EB) at 40×.

### In vivo tumor model

Thirty-two male athymic mice, taken from King Faisal Hospital and research center, Riyadh, KSA, were incarcerated in the contagion-free atmosphere (12 h light/dark cycle), and given food ad libitum. HCT116 cancer cells were grown in RPMI 1640 media and injected (1 × 10^6^, mixed with matrigel in an equivalent proportion [Collaborative Biomedical Products, Bedford, MA)] subcutaneously to mice. Tumor measurements began one-week post-injection. Tumor dimensions (length, width, and height) were quantified with Vernier caliper and their volume was calculated via ellipsoidal formula^72, 73^.

$${\text{Volume}}\,{\text{of}}\,{\text{tumor}} \; \left( {{\text{mm}}^{3} } \right)\, = \,\pi /6\, \times \,H \; \left( {{\text{mm}}} \right)\, \times \,L1 \; \left( {{\text{mm}}} \right)\, \times \,L2 \; \left( {{\text{mm}}} \right).$$

*H* is the tumor height, *L*1 and *L*2 is the long and short diameter respectively^74^.

When tumor volumes reached the dimension of 60–100 mm^3^, the mice were divided into four groups (*n* = 8). The trial was planned following the earlier procedure with trivial changes^75^.

Group 1: received distilled water.

Group 2: received 15 mg/kg ip CisPT and repeated dose 2 weeks later^76, 77^.

Group 3: administered 400 mg/kg AHE daily for 7 days than 4 doses per week for the next 2 weeks.

Group 4: administered 400 mg/kg AHE daily for 7 days before CisPT first injection than 4 doses per week for the next 2 weeks before the 2nd CisPT shot.

The size of the tumor mass was measured twice during the experiment. Food intake, water expenditure, and the body weight of animals were recorded twice a week. 7 days after the final administration of CisPt and AHE, mice were anesthetized with CO_2_ inhalation, and their blood was drawn in vacutainers and the kidneys were incised. Blood serum examined for blood urea nitrogen (BUN) and creatinine (sCr) levels, while kidney tissue was used to measure tissue enzyme concentration and lipid peroxidation intensities.

### Evaluation of oxidative trauma indicators

GSH levels were quantified using the previous methodology^24^. Lipid peroxidation level was evaluated by estimating thiobarbituric reactive elements via a commercial kit (Cayman Chemicals, Ann Arbor, MI).

### Statistical analysis

Data presented as mean ± SEM (*n* = 6 and *n* = 8). The statistical variances amid treatment groups were examined by one-way analysis of variance (ANOVA) along with Tukey’s test by Graph pad prism 5 software. The level of significance was set at *p* < 0.05.

### Ethics approval and consent to participate

This study involves rat and mice models and the experimental protocol for the use of animals was approved (Bch#0256) by the ethical board of Quaid-i-Azam University, Islamabad Pakistan.

## Supplementary Information

Supplementary Information 1.

Supplementary Information 2.

Supplementary Information 3.

Supplementary Information 4.

## Data Availability

The datasets used and/or analyzed during the current study are available from the corresponding author on reasonable request.

## Abbreviations

CisPT: Cisplatin
AHE: *Acacia hydaspica* Ethyl acetate extract
SOD: Superoxide dismutase
CAT: Catalase
POD: Peroxidase
QR: Quinone reductase
GPx: Glutathione peroxidase
γ-GT: Gamma glutamyl transferase
GST: Glutathione S transferase
GR: Glutathione reductase
H_2_O_2_: Hydrogen peroxide
NO: Nitric oxide
MDA: *Malondialdehyde*
WBCs: White blood cells
RBCs: Red blood cells
