# Supplementary material for: Acacia hydaspica R. Parker ethyl-acetate extract abrogates cisplatin-induced nephrotoxicity by targeting ROS and inflammatory cytokines
Source: Sci Rep. 2021 Aug 26;11:17248. doi: 10.1038/s41598-021-96509-y (PMC8390681; doi:10.1038/s41598-021-96509-y)
Supplement: Supplementary file 1 — Supplementary Information 1. [file 41598_2021_96509_MOESM1_ESM.pdf]

Acacia hydaspica R. Parker ethyl-acetate extract abrogates Cisplatin-induced nephrotoxicity by targeting ROS and inflammatory cytokines.

Tayyaba Afsar[1*], Suhail Razak[1*,] Dara Aldisi[1], Maria Shabbir[2,] Ali Almajwal[1], Abdulaziz Abdullah Al Khuraif[3], Mohammed Arshad[3]

Table 1: List of primers

Candidate gene	primer
**NF-κB p65**	F: 5′- ACACCTCTGCATATAGCGGC-3′<br>R: 5′- GGTACCCCCAGAGACCTCAT-3′
**IL-6**	F: 5′-ACCTGCTCCACTGCCTTGCT-3′<br>R: 5′-GGTTGCCAAGCCTTATCGGA-3/
