# Supplementary material for: Acacia hydaspica R. Parker ethyl-acetate extract abrogates cisplatin-induced nephrotoxicity by targeting ROS and inflammatory cytokines
Source: Sci Rep. 2021 Aug 26;11:17248. doi: 10.1038/s41598-021-96509-y (PMC8390681; doi:10.1038/s41598-021-96509-y)

Acacia hydaspica R. Parker ethyl-acetate extract abrogates Cisplatin-induced nephrotoxicity by targeting ROS and inflammatory cytokines.

Tayyaba Afsar[1*], Suhail Razak[1*,] Dara Aldisi[1], Maria Shabbir[2,] Ali Almajwal[1], Abdulaziz Abdullah Al Khuraif[3], Mohammed Arshad[3]

Supplementary file 2: Histological sections

Control

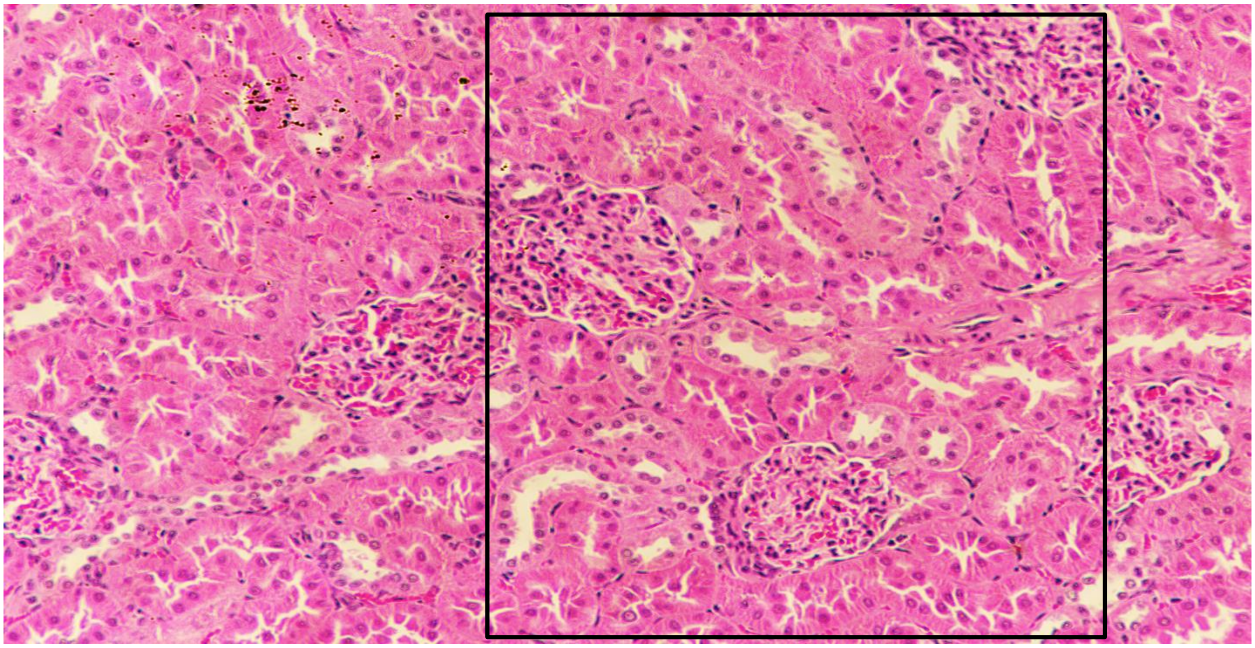

CisPT alone treatment view 1 20x

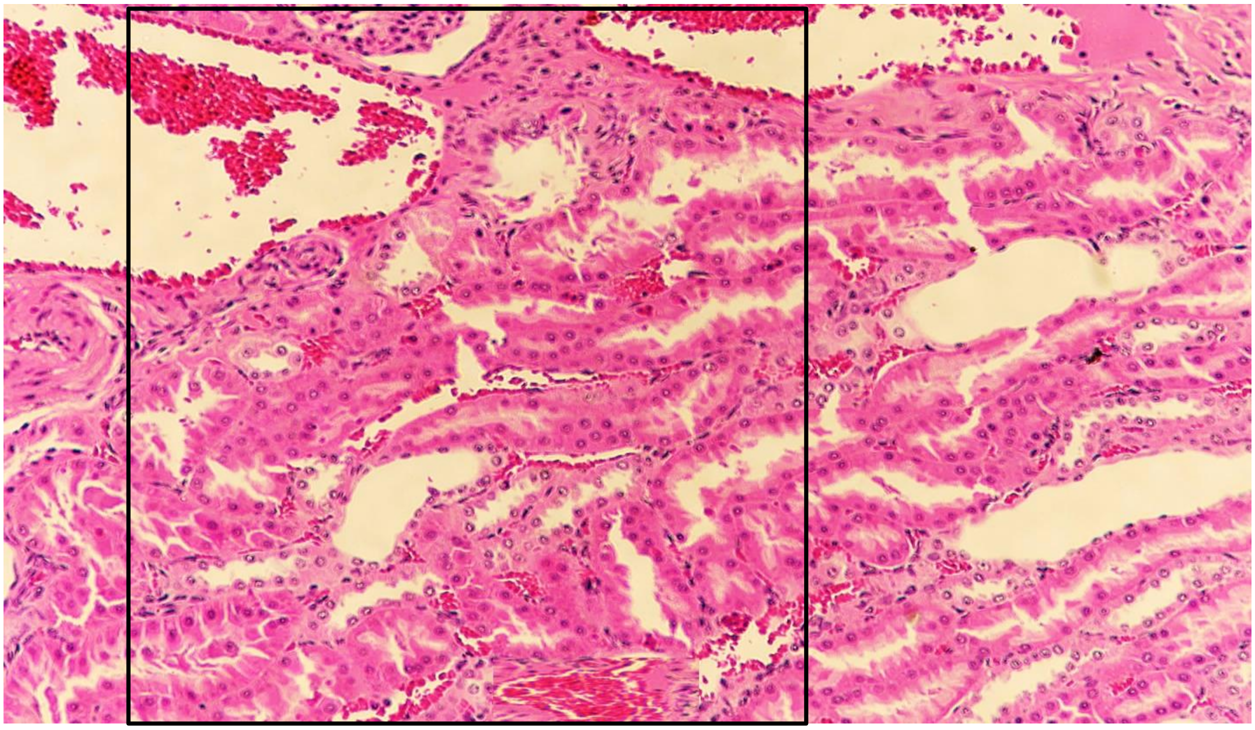

View 2 20x

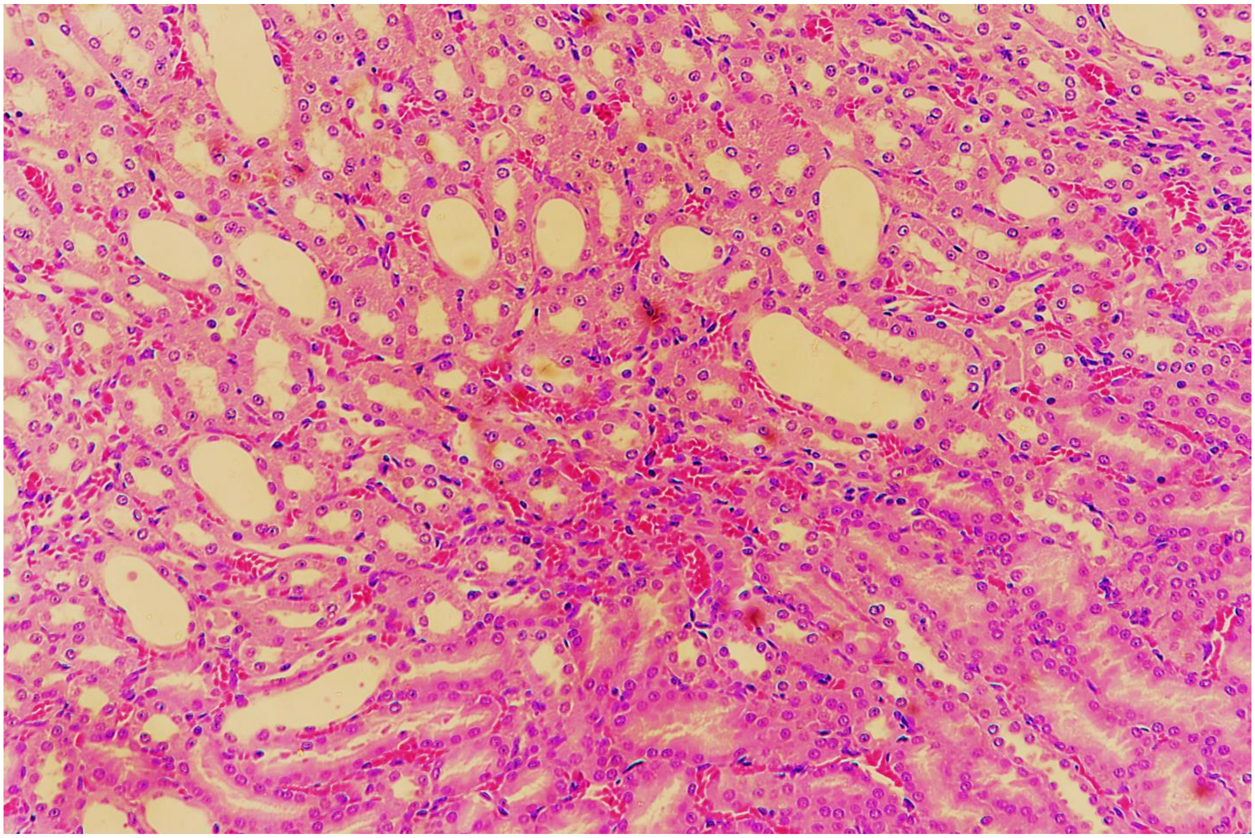

AHE alone (400 mg/kg bw) 20x

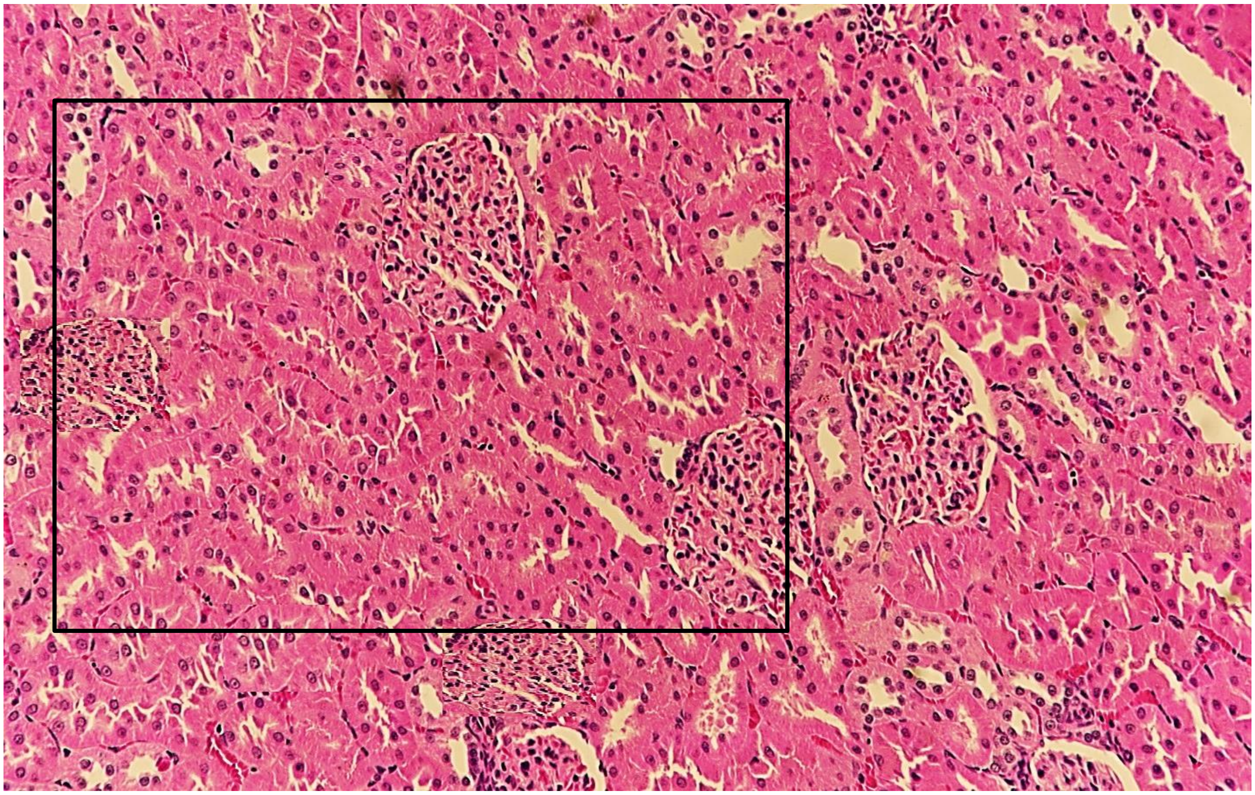

CisPt + AHE (400mg/kg bw post treated) 20x

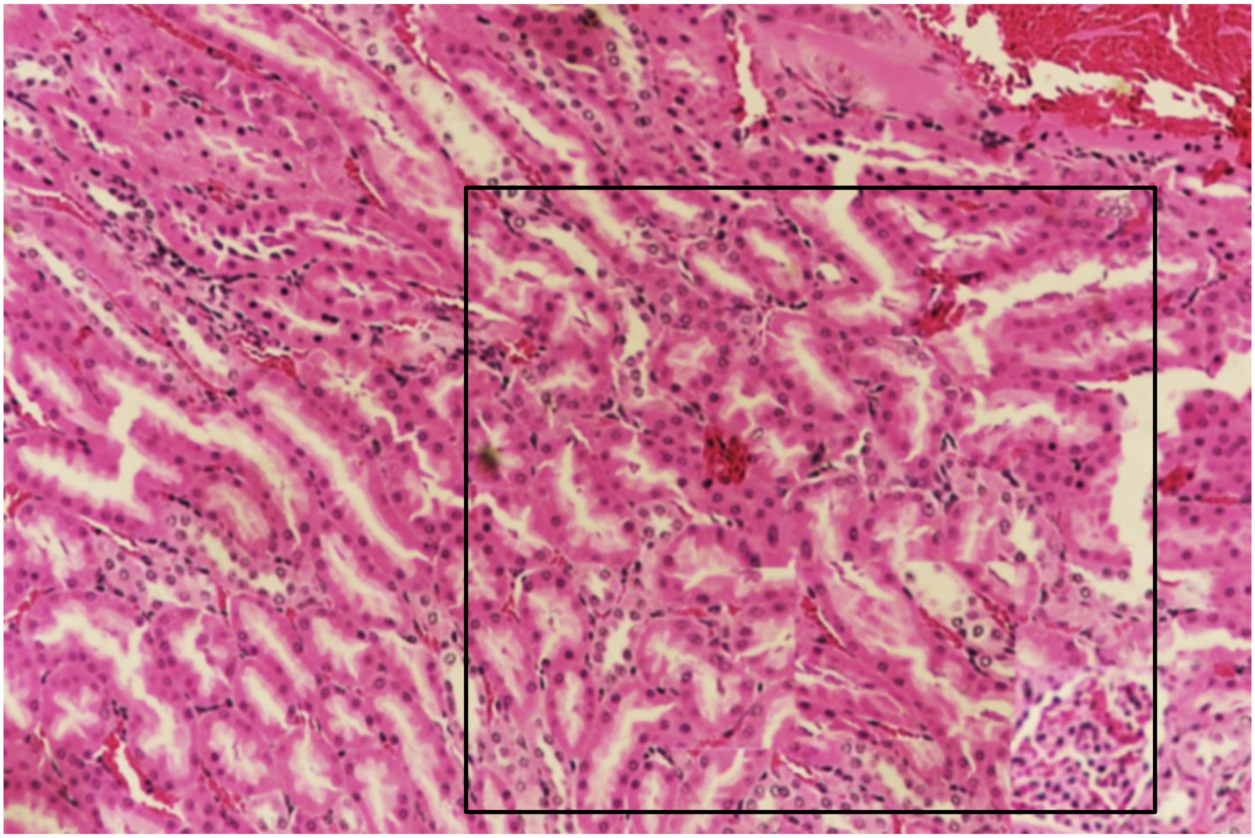

AHE (400mg/kg bw Pretreated) + CisPT 20x

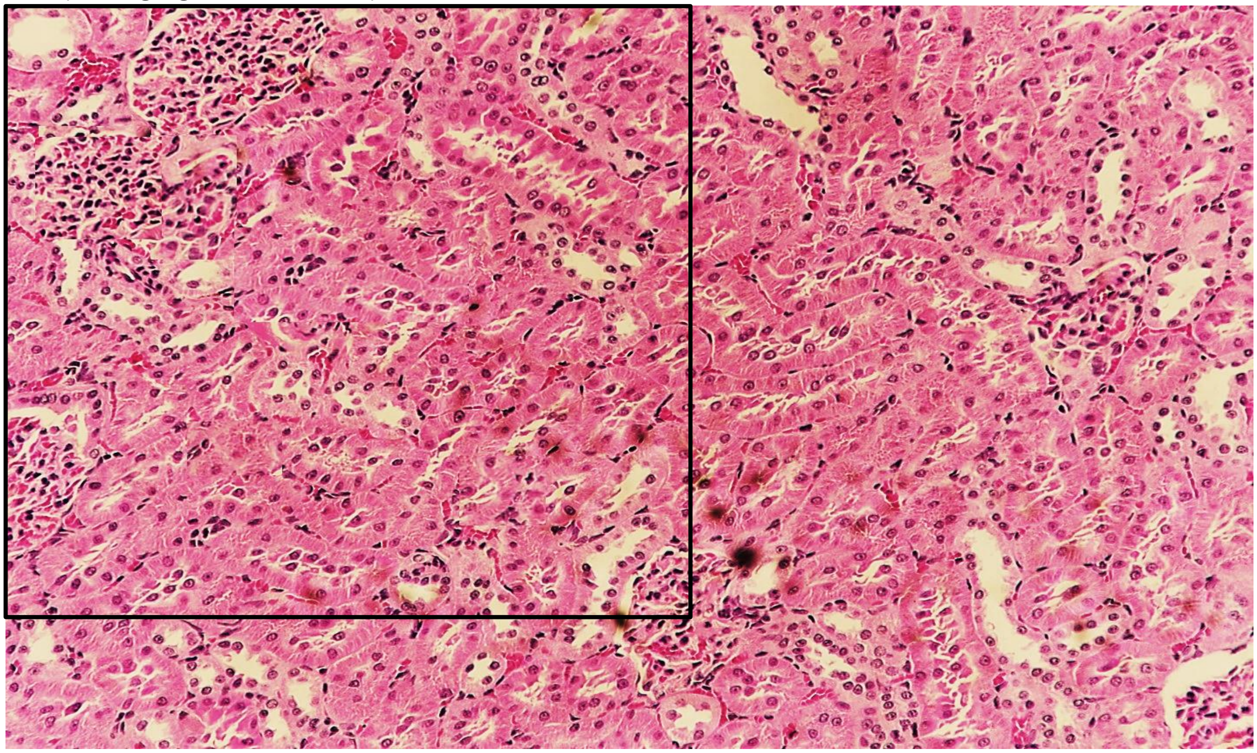

Silymarin +CisPT 20x

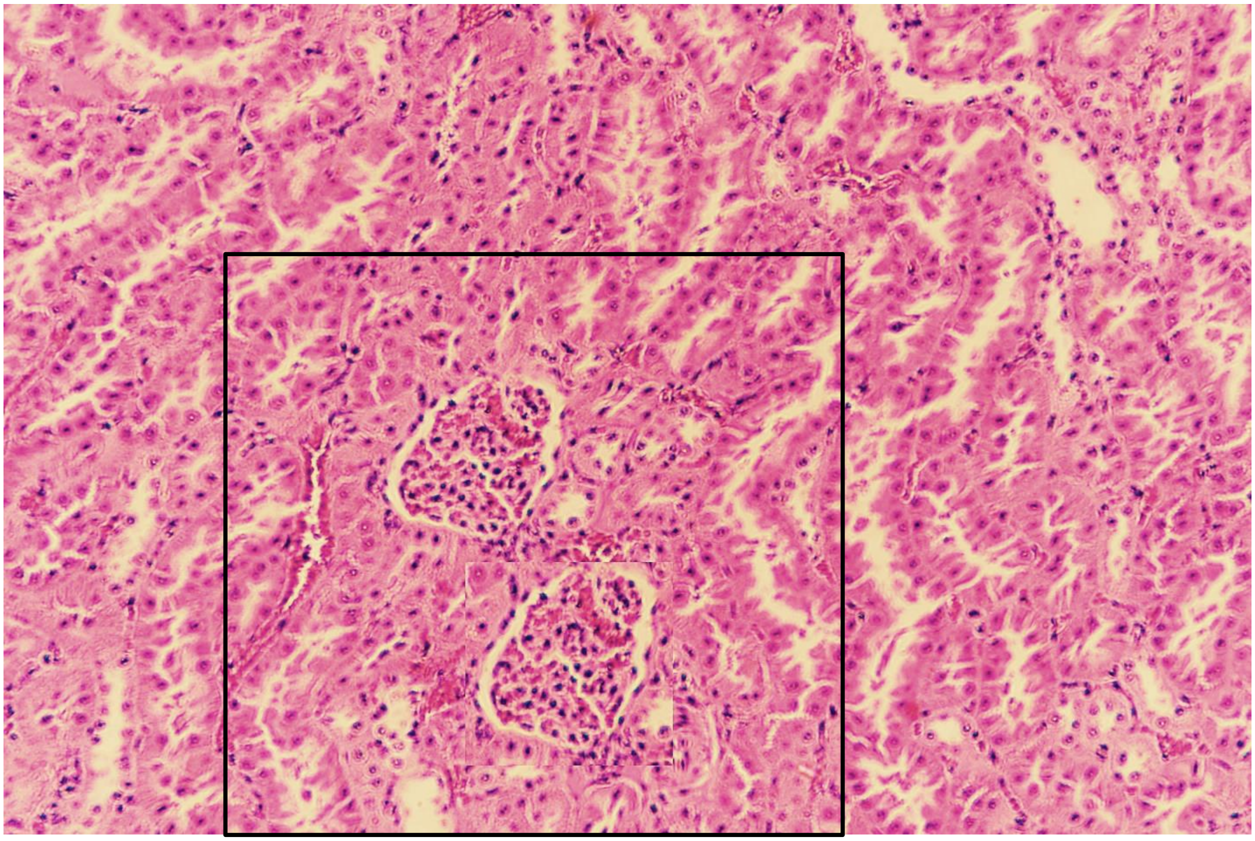

Supplement: Supplementary file 2 — Supplementary Information 2. [file 41598_2021_96509_MOESM2_ESM.pdf]
