# Supplementary material for: Acacia hydaspica R. Parker ethyl-acetate extract abrogates cisplatin-induced nephrotoxicity by targeting ROS and inflammatory cytokines
Source: Sci Rep. 2021 Aug 26;11:17248. doi: 10.1038/s41598-021-96509-y (PMC8390681; doi:10.1038/s41598-021-96509-y)
Supplement: Supplementary file 3 — Supplementary Information 3. [file 41598_2021_96509_MOESM3_ESM.pdf]

***Acacia hydaspica* R. Parker ethyl-acetate extract abrogates Cisplatin-induced nephrotoxicity by targeting ROS and inflammatory cytokines.**

Tayyaba Afsar[1*], Suhail Razak[1*,] Dara Aldisi[1], Maria Shabbir[2,] Ali Almajwal[1], Abdulaziz Abdullah Al Khuraif[3], Mohammed Arshad[3]

Supplementary S3: Table 2: Phytochemical composition of *Acacia hydaspica* ethyl acetate fraction (AHE)

| **Detection method** | **Metabolites** | **References** | **Biological activity/Chemo-preventive potential /References** |
|---|---|---|---|
| **Qualitative screening** | Tannin | (1) | Antioxidant, anticancer (2), Nehroprotective against CP, increase bioavailability of CP (3), inhibit CP–induced TBARS production in rat kidney (4). |
| | Steroids | | Antiinflammaorty (5, 6), anticancer (7), Nehroprotective against CP (8) |
| | Alkaloids | | Antiinflammaorty(5, 6), antioxidant, chemopreventive, anticancer (9), nephroprotective against CP-induced renal injury via inhibition of oxidative/nitrosative stress, inflammation, autophagy and apoptosis (10). |
| | Flavonoids | | Anti-inflammatory (5, 6, 11), antioxidant, anticancer, ameliorate cisplatin-induced nephrotoxicity via anti-apoptotic and anti-inflammatory effects (12). |
| | Coumarins | | Anti-inflammatory (13), antioxidant (14), anti-cancer (15). |
| | Terpenoids | | Antiinflammatiry [5, 6], Chemopreventive, anticancer[10] renoprotective against CP [11]. |
| **Quantitative Estimation** | *Flavonoids*:<br>129±1.32 TFC (mg rutin equivalent/g dry sample) | (1) | Antioxidant, anticancer, induced apoptosis, inhibit oxidative stress, flavonids exibit protection against CP induced nephrotoxicity (2), i.e. rutin a flavonoid effectively reduced the cisplatin-induced renal toxicity in albino rats by ameliorating serum kidney function markers, creatinine clearance, and renal malondialdehyde levels (16). |

| | | | |
|---|---|---|---|
| | *Phenolics*: 120.3±1.15 TPC (mg gallic acid equivalent/g dry sample) | | Antioxidant ,anticancer (17), anti-inflammatory, antitumour, anti-proliferative (18), Chemopreventive and anticancer activity of GSE in various cancers attribitued to the presence of poly phenolics constituents and their antioxidant potential (19). |
| **HPLC-DAD Screening using standard flavonoids** | Gallic acid (4.52 52.92 µg/100 mg dry powder) | (1) | Antioxidant, antimutagenic, chemopreventive (20), inhibit CP induce acculmalation of TBARS in renal tissues *in vitro* (4), modulate antioxidant status and prevent CP induce kidney demage in rats (21). |
| | Catechin (11.43 8648.0 µg/100 mg dry powder) | | Antioxidant , anticancer (22) , chemopreventive (23) |
| | Myricetin (17.08 34.60 µg/100 mg dry powder) | | Myricetin exhibited a protective against cisplatin-induced nephrotoxicity in rats due to its antioxidant and anti-inflammatory effects (24) phenolic compounds present in extracts justify their marked antioxidant activities. |
| **Isolation of pure bioactive compounds through bioassay guided fractionation and isolation.** | 7-*O*-galloylcatechin (187.5 mg/g) | (11, 25) | Antioxidant (26), anticancer, antiproliferation and apoptotic against breast and prostate cancer (25), prevented CP-induced oxidative stress, inflammation, and apoptosis (27). |
| | +Catechin (100 mg/g) | | Anticancer, antioxidant, proapoptotic (25) , nephroprotective ; renoprotective effect of catechin hydrate against gentamicin-induced nephrotoxicity might be mediated through its antioxidant and possible direct nephroprotective actions (28). |
| | Methyl gallate (37.5 mg/g) | | Anticancer, antioxidant (25, 29), prevent oxidative stress and DNA damage in renal cells via scavenging of intracellular reactive oxygen species (ROS), inhibition of lipid peroxidation and prevention of intracellular GSH depletion (30). |

TFC: Total flavonoid content, TPC: Total phenolic content. Phytoconstituents of *Acacia hydaspica* ethylacetate fractions derived from our lab previous investigations.

## References


1. Afsar T, Razak S, Khan MR, Mawash S, Almajwal A, Shabir M, et al. Evaluation of antioxidant, anti-hemolytic and anticancer activity of various solvent extracts of Acacia hydaspica R. Parker aerial parts. BMC complementary and alternative medicine. 2016;16.
2. Ojha S, Venkataraman B, Kurdi A, Mahgoub E, Sadek B, Rajesh M. Plant-Derived Agents for Counteracting Cisplatin-Induced Nephrotoxicity. Oxidative Medicine and Cellular Longevity. 2016;2016.
3. Tikoo K, Bhatt DK, Gaikwad AB, Sharma V, Kabra DG. Differential effects of tannic acid on cisplatin induced nephrotoxicity in rats. FEBS letters. 2007;581(10):2027-35.
4. Ayodele JA, Ayodeji AO, Oluwafemi TO, Sunday AO, Scholarstical A. Inhibitory effect of tannic acid and its derivative (gallic acid) against cisplatin–induced thiobarbituric acid reactive substances (TBARS) production in rat kidney–in vitro. International Journal of Advanced Research. 2015;3(1):116-26.
5. Ko H-H, Hung C-F, Wang J-P, Lin C-N. Antiinflammatory triterpenoids and steroids from Ganoderma lucidum and G. tsugae. Phytochemistry. 2008;69(1):234-9.
6. Beg S, Swain S, Hasan H, Barkat MA, Hussain MS. Systematic review of herbals as potential anti-inflammatory agents: Recent advances, current clinical status and future perspectives. Pharmacognosy reviews. 2011;5(10):120.
7. Salvador JA, Carvalho JF, Neves MA, Silvestre SM, Leitão AJ, Silva MMC, et al. Anticancer steroids: linking natural and semi-synthetic compounds. Natural product reports. 2013;30(2):324-74.
8. Yasuhiro K, Jiro U, Toyofumi U, Tetsuo Y, Joichi K. Prophylactic effect of methylprednisolone against cisplatin-induced nephrotoxicity in rats. Toxicology letters. 1993;66(3):281-5.
9. Lu J-J, Bao J-L, Chen X-P, Huang M, Wang Y-T. Alkaloids isolated from natural herbs as the anticancer agents. Evidence-Based Complementary and Alternative Medicine. 2012;2012.
10. Domitrović R, Cvijanović O, Pernjak-Pugel E, Škoda M, Mikelić L, Crnčević-Orlić Ž. Berberine exerts nephroprotective effect against cisplatin-induced kidney damage through inhibition of oxidative/nitrosative stress, inflammation, autophagy and apoptosis. Food and chemical toxicology. 2013;62:397-406.
11. Afsar T, Khan MR, Razak S, Ullah S, Mirza B. Antipyretic, anti-inflammatory and analgesic activity of Acacia hydaspica R. Parker and its phytochemical analysis. BMC complementary and alternative medicine. 2015;15(1):136.
12. Athira K, Madhana RM, Lahkar M. Flavonoids, the emerging dietary supplement against cisplatin-induced nephrotoxicity. Chemico-biological interactions. 2016;248:18-20.
13. Egan D, O'kennedy R, Moran E, Cox D, Prosser E, Thornes RD. The pharmacology, metabolism, analysis, and applications of coumarin and coumarin-related compounds. Drug metabolism reviews. 1990;22(5):503-29.

14. Al-Amiery AA, Al-Majedy YK, Kadhum AAH, Mohamad AB. Novel macromolecules derived from coumarin: synthesis and antioxidant activity. Scientific reports. 2015;5:11825.
15. Nasr T, Bondock S, Youns M. Anticancer activity of new coumarin substituted hydrazide–hydrazone derivatives. European journal of medicinal chemistry. 2014;76:539-48.
16. Sreedevi A, Bharathi K, Prasad K. Protective effect of rutin against cisplatin-induced nephrotoxicity in rats. Journal of Natural Remedies. 2010;10(2):144-51.
17. Zhou K, Raffoul JJ. Potential anticancer properties of grape antioxidants. Journal of oncology. 2012;2012.
18. Chen M, Meng H, Zhao Y, Chen F, Yu S. Antioxidant and in vitro anticancer activities of phenolics isolated from sugar beet molasses. BMC complementary and alternative medicine. 2015;15(1):313.
19. Kaur M, Agarwal C, Agarwal R. Anticancer and cancer chemopreventive potential of grape seed extract and other grape-based products. The Journal of nutrition. 2009;139(9):1806S-12S.
20. Badhani B, Sharma N, Kakkar R. Gallic acid: a versatile antioxidant with promising therapeutic and industrial applications. RSC Advances. 2015;5(35):27540-57.
21. Akomolafe SF, Akinyemi AJ, Anadozie SO. Phenolic Acids (Gallic and Tannic Acids) Modulate Antioxidant Status and Cisplatin Induced Nephrotoxicity in Rats. International scholarly research notices. 2014;2014.
22. Evacuasiany E, Ratnawati H, Liana LK, Widowati W, Maesaroh M, Mozef T, et al. Cytotoxic and antioxidant activities of catechins in inhibiting the malignancy of breast cancer. Oxidants and Antioxidants in Medical Science. 2014;3(2):141-6.
23. Shimizu M, Shirakami Y, Sakai H, Kubota M, Kochi T, Ideta T, et al. Chemopreventive potential of green tea catechins in hepatocellular carcinoma. International journal of molecular sciences. 2015;16(3):6124-39.
24. Hassan SM, Khalaf MM, Sadek SA, Abo-Youssef AM. Protective effects of apigenin and myricetin against cisplatin-induced nephrotoxicity in mice. Pharmaceutical Biology. 2017;55(1):766-74.
25. Afsar T, Trembley JH, Salomon CE, Razak S, Khan MR, Ahmed K. Growth inhibition and apoptosis in cancer cells induced by polyphenolic compounds of Acacia hydaspica: Involvement of multiple signal transduction pathways. Scientific reports. 2016;6:23077.
26. Zhao C, Li C, Liu S, Yang L. The galloyl catechins contributing to main antioxidant capacity of tea made from Camellia sinensis in China. The Scientific World Journal. 2014;2014.
27. Malik S, Suchal K, Bhatia J, Gamad N, Dinda AK, Gupta YK, et al. Molecular mechanisms underlying attenuation of cisplatin-induced acute kidney injury by epicatechin gallate. Laboratory investigation; a journal of technical methods and pathology. 2016;96(8):853-61.
28. Sardana A, Kalra S, Khanna D, Balakumar P. Nephroprotective effect of catechin on gentamicin-induced experimental nephrotoxicity. Clinical and experimental nephrology. 2015;19(2):178-84.

29. Kamatham S, Kumar N, Gudipalli P. Isolation and characterization of gallic acid and methyl gallate from the seed coats of Givotia rottleriformis Griff. and their anti-proliferative effect on human epidermoid carcinoma A431 cells. Toxicology Reports. 2015;2:520-9.
30. Hsieh T-J, Liu T-Z, Chia Y-C, Chern C-L, Lu F-J, Chuang M-c, et al. Protective effect of methyl gallate from Toona sinensis (Meliaceae) against hydrogen peroxide-induced oxidative stress and DNA damage in MDCK cells. Food and chemical toxicology. 2004;42(5):843-50.
