# Supplementary material for: Acacia hydaspica R. Parker ethyl-acetate extract abrogates cisplatin-induced nephrotoxicity by targeting ROS and inflammatory cytokines
Source: Sci Rep. 2021 Aug 26;11:17248. doi: 10.1038/s41598-021-96509-y (PMC8390681; doi:10.1038/s41598-021-96509-y)
Supplement: Supplementary file 4 — Supplementary Information 4. [file 41598_2021_96509_MOESM4_ESM.pdf]

Title: ***Acacia hydaspica*** **R. Parker ethyl-acetate extract abrogates Cisplatin-induced nephrotoxicity by targeting ROS and inflammatory cytokines.**

Tayyaba Afsar[1*], Suhail Razak[1*,] Dara Aldisi[1], Maria Shabbir[2,] Ali Almajwal[1], Abdulaziz Abdullah Al Khuraif[3], Mohammed Arshad[3]

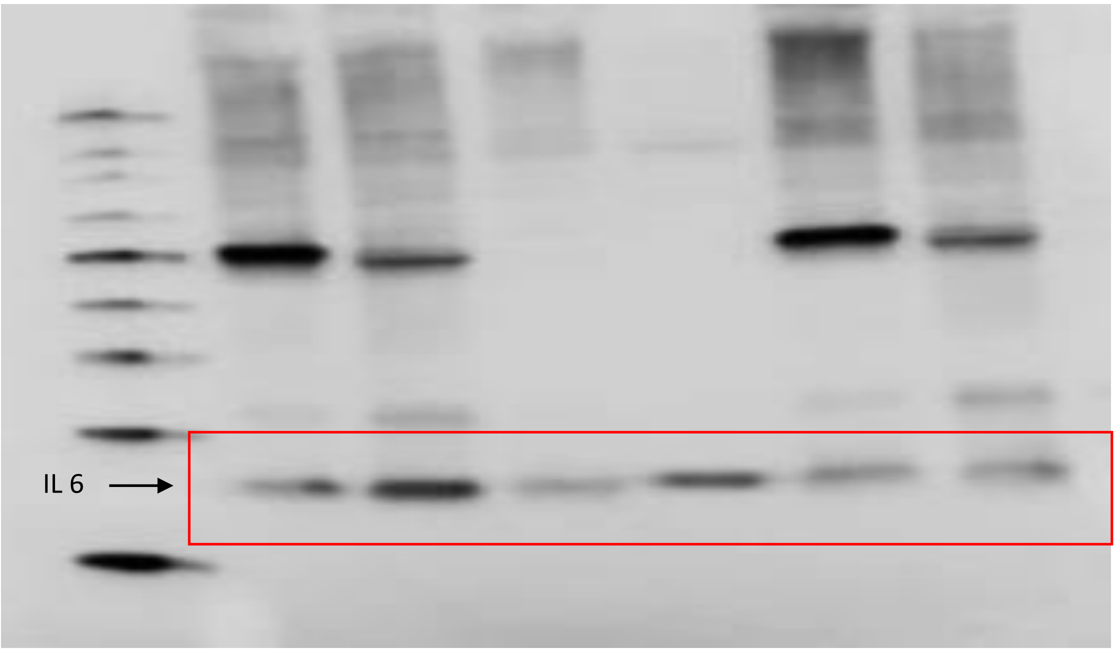

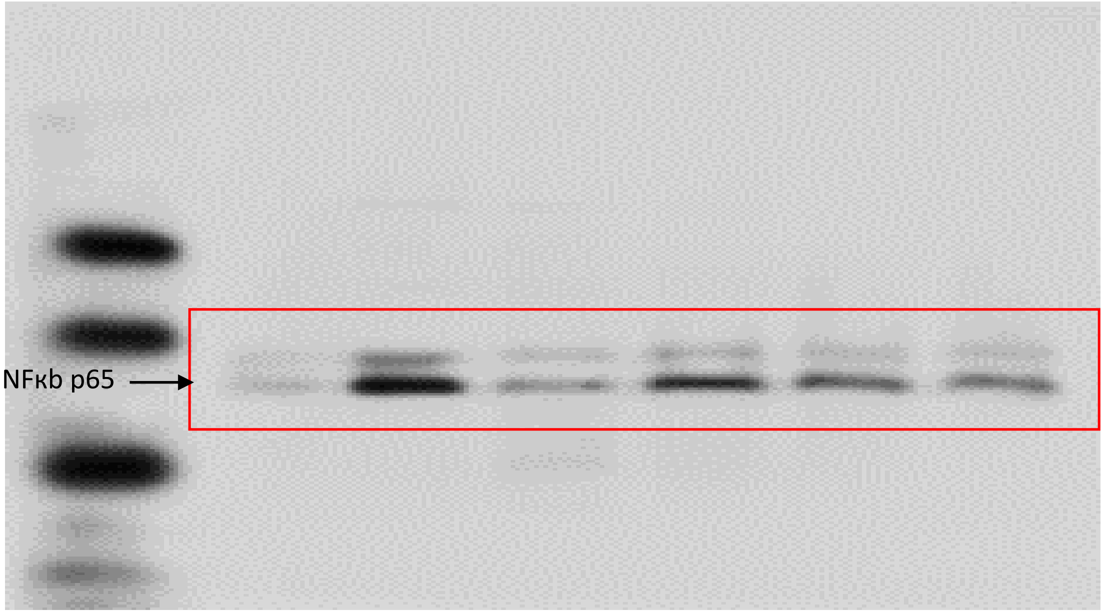

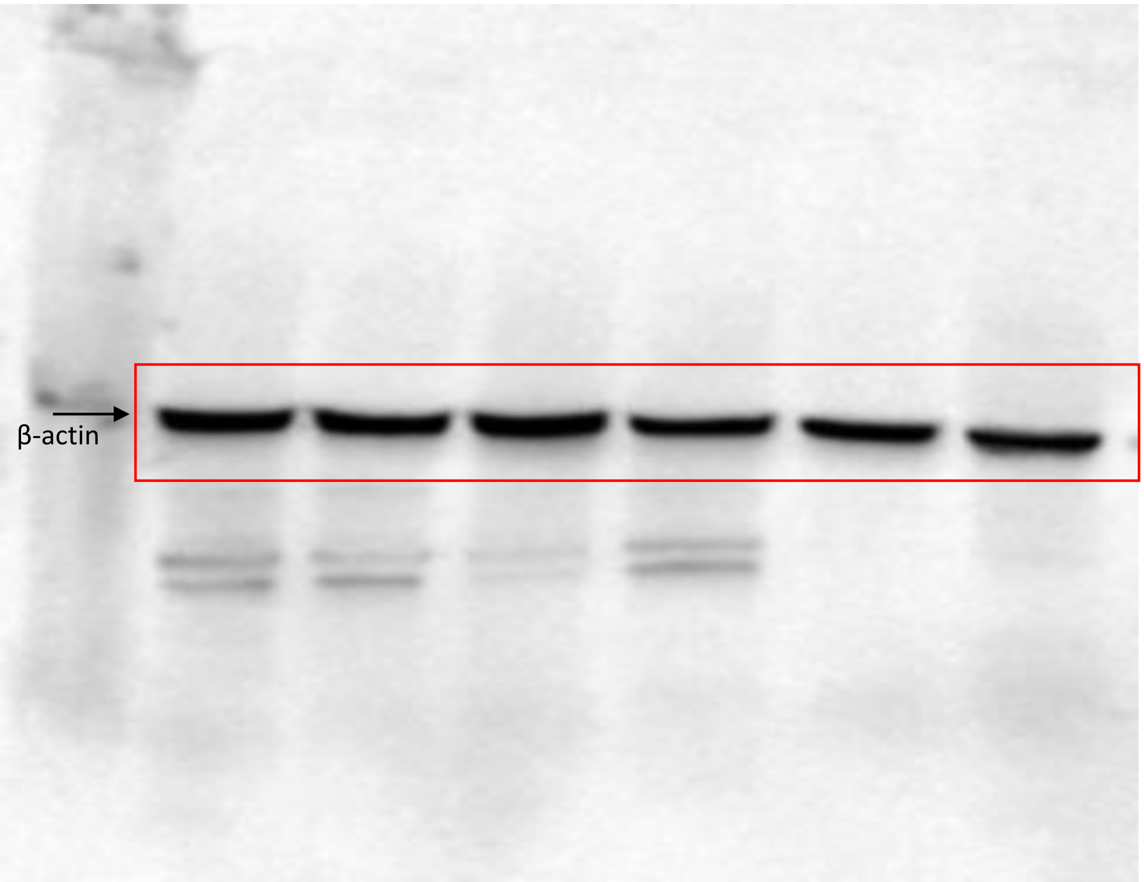
β-actin
